# Aspartate Metabolism‐Driven Gut Microbiota Dynamics and RIP‐Dependent Mitochondrial Function Counteract Oxidative Stress

**DOI:** 10.1002/advs.202404697

**Published:** 2025-01-28

**Authors:** Shunshun Jin, Jian Wu, Chenyu Wang, Yiwen He, Yulong Tang, Le Huang, Hui Zhou, Di Liu, Ziping Wu, Yanzhong Feng, Heshu Chen, Xinmiao He, Guan Yang, Can Peng, Jiazhang Qiu, Tiejun Li, Yulong Yin, Liuqin He

**Affiliations:** ^1^ Hunan Provincial Key Laboratory of Animal Intestinal Function and Regulation Hunan international joint laboratory of Animal Intestinal Ecology and Health Laboratory of Animal Nutrition and Human Health College of Life Sciences Hunan Normal University Changsha 410081 China; ^2^ Key Laboratory of Agro‐ecological Processes in Subtropical Region Institute of Subtropical Agriculture Chinese Academy of Sciences Hunan Provincial Key Laboratory of Animal Nutritional Physiology and Metabolic Process Changsha 410125 China; ^3^ Department of Animal Science University of Manitoba Winnipeg Manitoba R3T2N2 Canada; ^4^ Agricultural and Food Economics Queen's University Belfast Northern Ireland BT95PX UK; ^5^ Heilongjiang Academy of Agricultural Sciences Harbin 150086 China; ^6^ Department of Infectious Diseases and Public Health City University of Hong Kong Kowloon Hong Kong SAR 999077 China; ^7^ State Key Laboratory for Diagnosis and Treatment of Severe Zoonotic Infections Disease Key Laboratory for Zoonosis Research of the Ministry of Education College of Veterinary Medicine Jilin University Changchun 130025 China; ^8^ Yuelushan Laboratory No. 246 Hongqi Road, Furong District Changsha 410128 China

**Keywords:** aspartate, microbiota, oxidative stress, RIP pathway

## Abstract

Aspartate (Asp) metabolism‐mediated antioxidant functions have important implications for neonatal growth and intestinal health; however, the antioxidant mechanisms through which Asp regulates the gut microbiota and influences RIP activation remain elusive. This study reports that chronic oxidative stress disrupts gut microbiota and metabolite balance and that such imbalance is intricately tied to the perturbation of Asp metabolism. Under normal conditions, in vivo and in vitro studies reveal that exogenous Asp improves intestinal health by regulating epithelial cell proliferation, nutrient uptake, and apoptosis. During oxidative stress, Asp reduces *Megasphaera* abundance while increasing *Ruminococcaceae*. This reversal effect depends on the enhanced production of the antioxidant eicosapentaenoic acid mediated through Asp metabolism and microbiota. Mechanistically, the application of exogenous Asp orchestrates the antioxidant responses in enterocytes via the modulation of the RIP3‐MLKL and RIP1‐Nrf2‐NF‐κB pathways to eliminate excessive reactive oxygen species and maintain mitochondrial functionality and cellular survival. These results demonstrate that Asp signaling alleviates oxidative stress by dynamically modulating the gut microbiota and RIP‐dependent mitochondrial function, providing a potential therapeutic strategy for oxidative stress disease treatment.

## Graphical abstract

This study examines the role of aspartate (Asp) metabolism in regulating gut microbiota and oxidative stress in piglets. Through interaction with the RIP1‐RIP3‐MLKL and RIP1‐Nrf2‐NF‐κB signaling pathways, Asp improves mitochondrial function and enhances the production of the antioxidant eicosapentaenoic acid (EPA), counteracting oxidative stress. Findings reveal Asp as a potential therapeutic approach for oxidative stress‐related intestinal health issues.

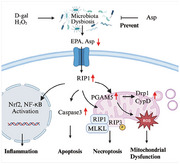

## 1 Introduction

Oxidative stress represents a substantial challenge to intestinal integrity and function in humans and animals. Infants and young animals with chronic oxidative stress exhibit wasting (impaired growth performance), stunting (reduced feed intake), deficiencies in development and immunity, and disrupted metabolism.^[^ ^1^, ^2^, ^3^ ^]^ Numerous studies have indicated that inhibiting mitochondrial oxidation, which produces excess reactive oxygen species (ROS), is essential for understanding and addressing diseases caused by chronic oxidative damage.^[^ ^4^, ^5^, ^6^, ^7^ ^]^ As a functional amino acid, aspartate (Asp) is oxidized for energy and regulates mitochondrial metabolism.^[^ ^8^ ^]^ It has been reported that in endothelial cells, inhibition of the electron transport chain (ETC) leads to reduced cell proliferation as well as decreased Asp levels, but Asp supplementation alone is not sufficient to rescue the impaired cell growth caused by ETC deficiency.^[^ ^9^ ^]^ This highlights a correlation between mitochondrial dynamics and Asp metabolism. Furthermore, clinical trials have shown that exogenous Asp alleviates oxidative stress caused by metabolic diseases, as evidenced by a significant increase in antioxidant enzyme activity and mitochondrial ATP levels.^[^ ^10^, ^11^ ^]^ These imply that Asp metabolism may affect the function and differentiation of mitochondria differently by regulating ROS levels in different states.

The gut microbiome and metabolism are intricately linked to the host immune responses to oxidative stress through the gut‐liver axis, as evidenced in several studies.^[^ ^12^, ^13^, ^14^ ^]^ Notably, alterations in the gut microbiota of mice on a high‐fat diet, such as a decrease in lactic acid bacterium and an increase in *Enterobacter* and *Escherichia*, are strongly linked to oxidative stress. This imbalance leads to mitochondrial dysfunction and intestinal epithelial cell apoptosis.^[^ ^15^ ^]^ Previous report has indicated that pretreatment with Asp effectively controls the antioxidant system by reducing lipid peroxidation in myocardial infarction models in rats.^[^ ^16^ ^]^ Moreover, dietary supplementation with Asp enhances intestinal integrity and energy status in piglets, demonstrating its potential to improve gut health in animal models.^[^ ^17^ ^]^ Additionally, the gut microbiota production of short‐chain fatty acids, including acetate, influences the immune response. Acetate, a result of Asp fermentation, not only modulates the formation of Asp‐derived antimicrobial peptides but also reduces macrophage tumor necrosis factor‐alpha (TNF‐α) secretion and activates regulatory T cells in the pancreas.^[^ ^18^, ^19^, ^20^ ^]^ These results suggest that Asp may participate in improving the gut microbiota and mitochondrial function to enhance the antioxidant capacity; however, the mechanism requires further exploration.

Receptor‐interacting protein kinase‐1 (RIP1) is the main regulatory factor in cell survival and death.^[^ ^21^, ^22^, ^23^ ^]^ RIP1 can induce cell apoptosis via the recruitment of cysteinyl aspartate‐specific protease 8, while also modulating necroptosis through the phosphorylation of receptor‐interacting protein kinase‐3 (RIP3), thus activating mixed lineage kinase domain‐like (MLKL).^[^ ^24^, ^25^, ^26^ ^]^ Some studies have shown that suppressing RIP1 expression in rat embryonic cardiac myocytes can stimulate the Akt/Nrf2 pathway, thereby mitigating mitochondrial damage and autophagy.^[^ ^27^ ^]^ Notably, RIP3 activation in the intestinal epithelial cells initiates cell death, leading to spontaneous colitis or ileitis in mice.^[^ ^28^ ^]^ Moreover, inhibiting RIP1 ubiquitination in BV2 cells has been linked to NF‐κB deactivation, attenuating TNF‐α‐induced neuroinflammation.^[^ ^29^ ^]^ A recent report has shown that upon treatment with the Asp metabolite glutamine, RIP3 can be phosphorylated and targeted by the pyruvate dehydrogenase complex (PDC), promoting ROS generation and aerobic respiration in the context of TNF‐induced necroptosis.^[^ ^30^ ^]^ Under starvation conditions, RIP1 deficiency leads to an accumulation of Asp in the mouse brain and heart cells, fueling ATP production and directly inhibiting autophagy.^[^ ^31^ ^]^ Hence, we speculate that the RIP pathway might be a bridge for Asp metabolism to regulate oxidative damage. However, the specific mechanisms underlying the antioxidant function of the Asp‐mediated RIP pathway have not yet been studied.

Here, we investigated the mechanisms associated with the role of Asp in mediating the gut microbiota and RIP‐dependent mitochondrial function in a pig model of oxidative stress. We demonstrated that the gut microbes of piglets were slowly disordered as oxidative stress persisted and that gut microbiota dysbiosis further aggravated the progress of oxidative damage and Asp metabolism. Both in vivo and in vitro results confirmed that exogenous Asp alleviated oxidative damage and enhanced mitochondrial function and cell proliferation, wherein Asp‐mediated gut microbiota‐enhanced production of eicosapentaenoic acid (EPA) by interacting with RIP3‐MLKL and RIP1‐Nrf2‐NF‐κB pathway. Our findings demonstrate that Asp metabolism dynamically modulates gut microbiota and mitochondrial function via the RIP pathway, consequently protecting against oxidative damage. This may provide a basis for designing therapeutic strategies against oxidative stress‐related diseases.

## 2 Results

### 2.1 Oxidative Stress Reduced Growth Development and Altered the Intestinal Microflora of Weaned Piglet Model

**Figure 1:**
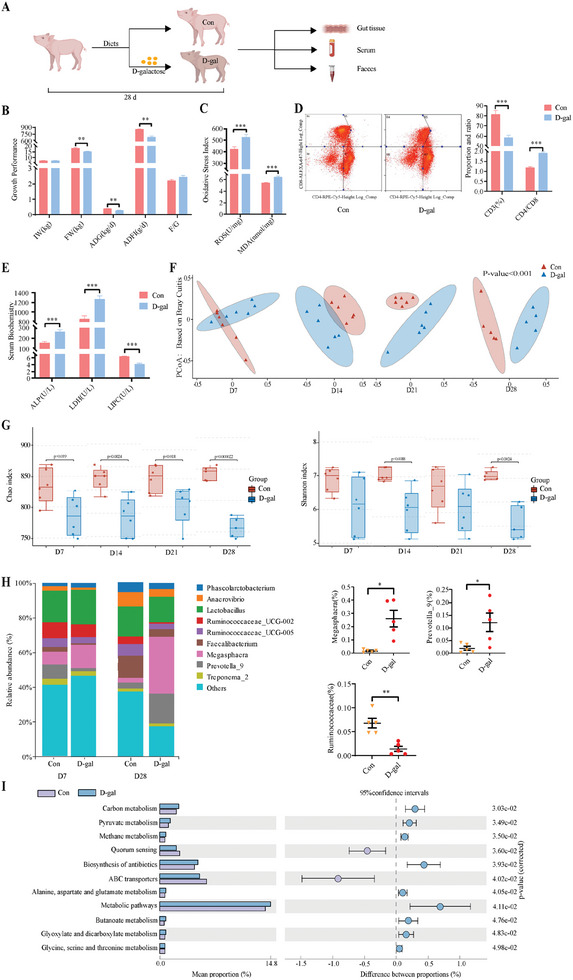
Growth performance and intestinal microflora in oxidative stress piglet model. A) The protocol shows the modeling of oxidative stress using dietary addition of D‐gal (10 g kg^−1^) for an experimental period lasting 28 days. B) Initial weight (IW), final weight (FW), average daily gain (ADG), average daily feed intake (ADF), and feed‐to‐weight ratio (F/G) of Con and D‐gal groups on day 28 (n = 6 per group). C) Concentrations of reactive oxygen species (ROS) and malondialdehyde (MDA) in serum (n = 6 per group). D) Blood T cell proportion by flow cytometry (n = 4 per group). E) Serum alkaline phosphatase (ALP), lactate dehydrogenase (LDH), and hepatic lipase (LIPC) concentrations (n = 6 per group). F) Feces were categorized by principal co‐ordinate analysis to demonstrate differences in species diversity between samples. The closer the samples are to each other on the co‐ordinate plot, the greater the similarity (n = 5 – 6 per group). G) Feces were evaluated for the Alpha Diversity Index using QIIME2 software. Chao1 was used to measure species abundance. Shannon was used to measure species diversity (n = 5 – 6 per group). H) Species abundance map at the genus level. One color represents one species, and the length of the color block indicates the proportion of relative abundance occupied by the species (only the top ten species at the abundance level are shown; Day 7: n = 6 per group, Day 28: n = 5 per group). I) The use of PICRUSt2 software employs species annotation of the feature sequences to be predicted with the phylogenetic tree already available in the software, and the use of the IMG microbial genome data for the output of functional information and thus inferring the functional gene composition in the samples, so as to analyze the differences in function between different samples or subgroups (n = 6 per group). Statistics: Data are presented as mean ± SEM (*p < 0.05, **p < 0.01, ***p < 0.001). Test: Data were analyzed using unpaired two‐tailed unpaired Student's *t*‐test, except for the data in H (*Megasphaera* and *Prevotella_9*), which were analyzed using an unpaired *t*‐test with Welch's correction.

D‐galactose (D‐gal) was chosen to establish a chronic oxidative stress piglet model to analyze the development of oxidative stress‐related intestinal diseases (**Figure** **1**A).^[^ ^32^ ^]^ Administration of D‐gal increased serum ROS and malondialdehyde (MDA) levels while it decreased the Final body weight (FW), the average daily gain (ADG), and average daily feed intake (ADFI) (Figure 1B,C), suggesting that the weaned piglet model of chronic oxidative stress was successfully established. Flow cytometry showed a reduction in CD3^+^ percentage and an increased CD4^+^/CD8^+^ ratio following D‐gal treatment. Serum biochemical analysis showed higher alkaline phosphatase (ALP) and lactate dehydrogenase (LDH) activities and a decrease in hepatic lipase (LIPC) activity under oxidative stress (Figure 1D,E). These results suggest that D‐gal‐induced chronic oxidative stress inhibits piglet growth and immune function and causes metabolic dysregulation over time.

We analyzed the gut microbiota of weaned piglets using 16S rRNA sequencing to determine the impact of chronic oxidative stress. Non‐weighted principal co‐ordinate analysis revealed a divergence in microbial composition between control and D‐gal groups from d1 to d28, with significant deference emerging on d28 (Figure 1F). The alpha diversity index, indicating species richness and diversity, decreased under oxidative stress, with notable declines in species abundance and in diversity on d14 and d28 (Figure 1G). At the genus level, D‐gal treatment significantly increased *Megasphaera* and *Prevotella_9 colonies* but decreased *Ruminococcaceae* (UCG‐002 and UCG‐005) (Figure 1H and Figure S1, Supporting Information). By linking SILVA classification with the KEGG database via the QIIME platform, we predicted that altered microbiota affected mitochondrial respiratory metabolism, protein and amino acid transport, antibiotic synthesis, and the metabolism of various amino acids such as alanine, aspartate, glutamate, glycine, serine, and threonine (Figure 1I). These results suggest that oxidative stress dynamically alters the intestinal microbiota profile of weaned piglets.

### 2.2 Intestinal Microbial and Metabolic Disorders Induced by Oxidative Stress Were Associated With Insufficient Supply of Endogenous Aspartate

**Figure 2:**
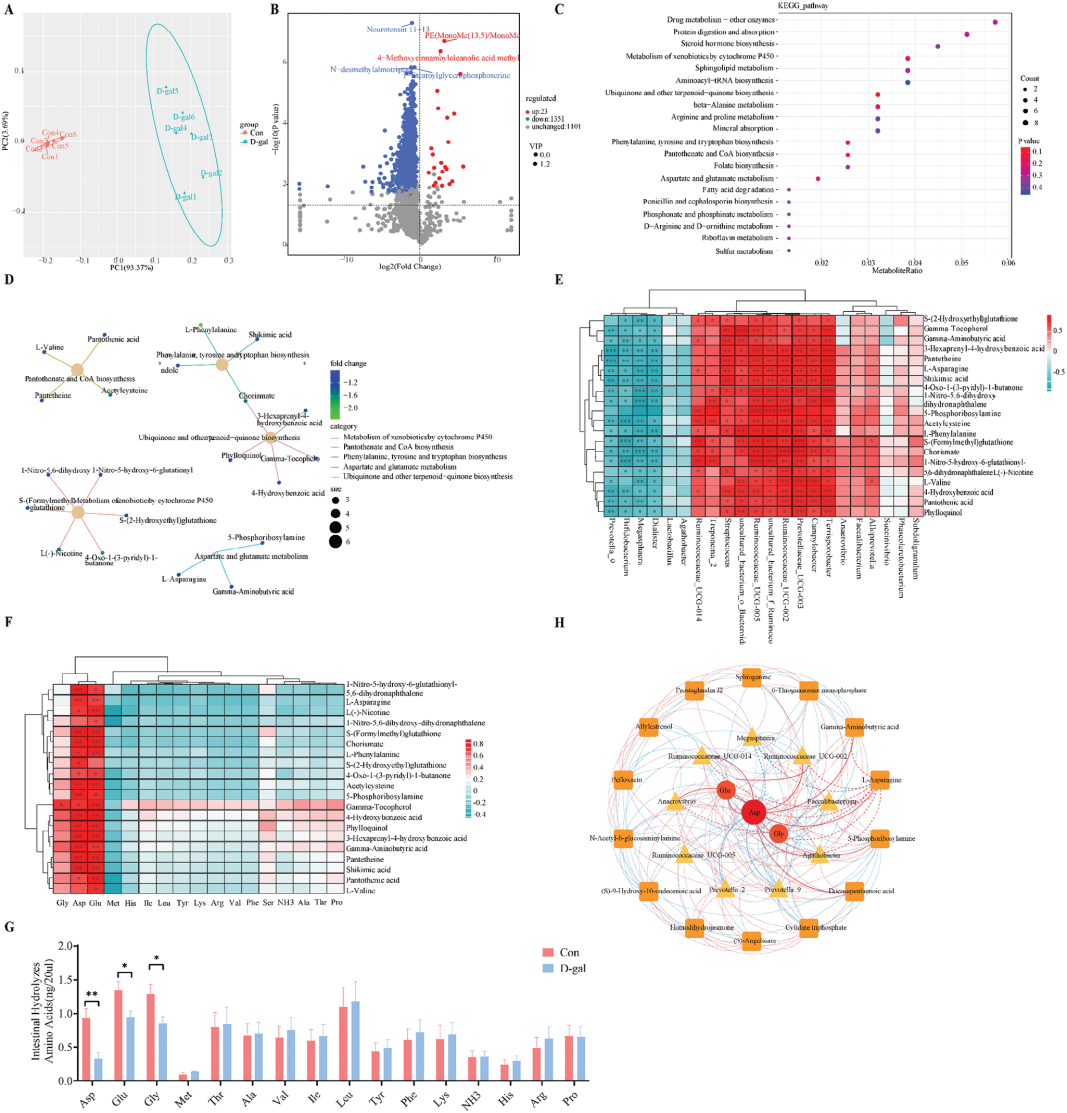
Effects of oxidative stress on fecal metabolites and amino acids metabolism. A) The principal component analysis demonstrated the overall metabolic differences between the samples in each group and the magnitude of variability between the samples within the groups. B) Volcano Plot showing the difference in metabolite expression levels in the two groups and the statistical significance of the difference (VIP: Variable Importance in Projection). Where each point represents a metabolite, the horizontal co‐ordinate represents the fold change of the group comparing each substance (taking logarithms with a base of 2), and the vertical co‐ordinate represents the *p*‐value of the unpaired two‐tailed Student's *t*‐test (taking logarithms with a base of 10). C) The annotation results of the differential metabolite KEGG were enriched and analyzed using clusterProfiler by choosing the hypergeometric test. D) Differential metabolite localization map (top five). The yellowish nodes in the figure are pathways, and the small nodes connected to them are specific metabolites annotated to that pathway. E) Heat map of Pearson correlation coefficients between differential microorganisms and metabolites. F) Pearson correlation coefficients between differential metabolites and amino acids. G) Amino acid concentrations in the digesta (Data are presented as mean ± SEM). H) Ternary multi‐dimensional analysis of correlations between amino acids, microorganisms, and metabolites. Statistics: n = 6 per group. Test: Data were analyzed using unpaired two‐tailed Student's *t*‐test, except for the data in panel E and F, which were analyzed using Pearson correlation. *p < 0.05, **p < 0.01, ***p < 0.001.

Our fecal metabolomics analysis showed a distinct separation in metabolite profiles between the control and D‐gal‐treated groups, suggesting that D‐gal alters the metabolome (**Figure** **2**A). There were 23 upregulated and 1351 downregulated metabolites in the D‐gal group compared to the control group (Figure 2B). ClusterProfiler analysis identified the KEGG pathways with differentially abundant metabolites were enriched. The pathways most impacted included xenobiotics metabolism by cytochrome P450, biosynthesis of pantothenate, CoA, phenylalanine, tyrosine, tryptophan, ubiquinone, and other terpenoid‐quinone (Figure 2C). Essential differential metabolites such as L‐asparagine, 5‐phosphoribosylamine, gamma‐aminobutyric acid, chorismate, and L‐phenylalanine were identified within these pathways (Figure 2D). The association analysis heat map indicated an extremely strong correlation between the main differential metabolites and differential microflora: *Ruminococcaceae*_UCG‐005 was positively correlated with 21 differential metabolites, while *Megasphaera and Prevotella_9* were negatively correlated with 20 and 19 differential metabolites, respectively (Figure 2E). Significant decreases in Asp, glutamate (Glu), and glycine (Gly) concentrations were observed in the D‐gal group, with Asp and Glu exhibiting an extremely strong positive correlation with most differential metabolites (Figure 2F,G). The ternary analysis of Asp, Glu, and Gly with different microorganisms and metabolites found a positive correlation between the changes in Asp, Glu, and Gly. Furthermore, the correlation of Asp with diverse microorganisms and metabolites was stronger than that of Glu and Gly (Figure 2H). Based on these observations, we hypothesize that aspartate might modulate intestinal microbiota and metabolism, particularly under oxidative stress conditions. We further postulate that oxidative stress may lead to increased consumption of endogenous Asp, potentially resulting in an insufficient supply, underscoring the need for further studies to conclusively determine the extent of Asp's involvement and the precise mechanisms by which oxidative stress impacts Asp levels.

### 2.3 Exogenous Aspartate Improved the Growth Performance of Weaned Piglets

**Figure 3:**
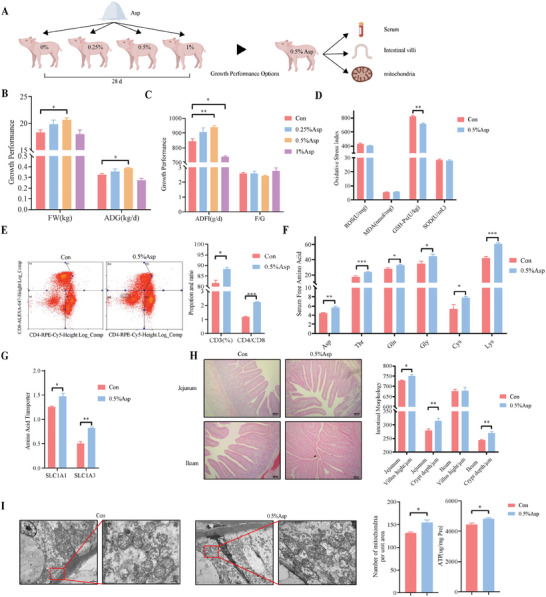
Effects of dietary aspartate supplementation on the growth of piglets under non‐stress conditions. A) During the 28‐day experimental period, different subgroups were established to be fed with different levels of Asp (0%, 0.25%, 0.5%, 1.0%) to screen the optimal concentration, and the best growth performance was selected for further testing. B, C) Growth performance (n = 6 per group). D) Serum antioxidant indexes (n = 3 per group). E) Blood T cell proportion and ratio (n = 3 per group). F) Serum‐free amino acid contents (only significant differences shown, n = 6 per group). G) Intestinal amino acid transporter expression (n = 6 per group). H) Light microscopy was used to measure villus height and crypt depth (Scale bar:100 µm, n = 6 per group). I) Mitochondrial morphology and number were observed and statistically analyzed using a JEM‐1400PLUS transmission electron microscope (Scale bar:1 µm and 500 nm) as well as the intestinal ATP content (n = 3 per group). Statistics: Data are presented as mean ± SEM. Test: Data were analyzed using unpaired two‐tailed Student's *t*‐test, except for the data in panel B and C, which were analyzed using a one‐way ANOVA with Dunnett's multiple comparison test. *p < 0.05, **p < 0.01, ***p < 0.001.

We established piglet diet groups with 0%, 0.25%, 0.5%, and 1.0% Asp concentration to determine the optimal Asp under normal physiological conditions (**Figure** **3**A). The results showed that adding 0.5% Asp to the daily diet significantly increased the FW and ADFI of piglets, whereas 1% Asp supplementation decreased the ADFI (Figure 3B,C), highlighting the need for precise Asp dosing due to the potential growth impediments form excess. A 0.5% Asp dietary supplementation alone had no significant effect on the oxidative stress indicators ROS, MDA, and superoxide dismutase (SOD) in serum, but it decreased the activity of glutathione peroxidase (GSH‐Px) (Figure 3D). Regarding immunity, the flow cytometry analysis showed that Asp supplementation increased the CD3^+^ percentage and CD4^+^/CD8^+^ ratio in the blood (Figure 3E). The expression of the intestinal Asp transporter proteins SLC1A1 and SLC1A3 increased. The levels of free amino acids such as Asp, Thr, Glu, Gly, Cys, and Lys in the serum were also increased, indicating boosted Asp transport and metabolism, consequently promoting the synthesis of other amino acids (Figure 3F,G). Histological examinations of the intestine showed that Asp remarkably increased jejunal villus height and intestinal ATP content, with electron microscopy displaying a higher mitochondrial density (Figure 3H,I). These results indicated that adding 0.5% Asp to the diet improves growth performance and intestinal health by enhancing immunity and promoting Asp metabolism, which is involved in mitochondrial energy metabolism.

### 2.4 Aspartate Promoted Cell Proliferation and Mitochondrial Respiratory Metabolism in IPEC‐J2 Cells

**Figure 4:**
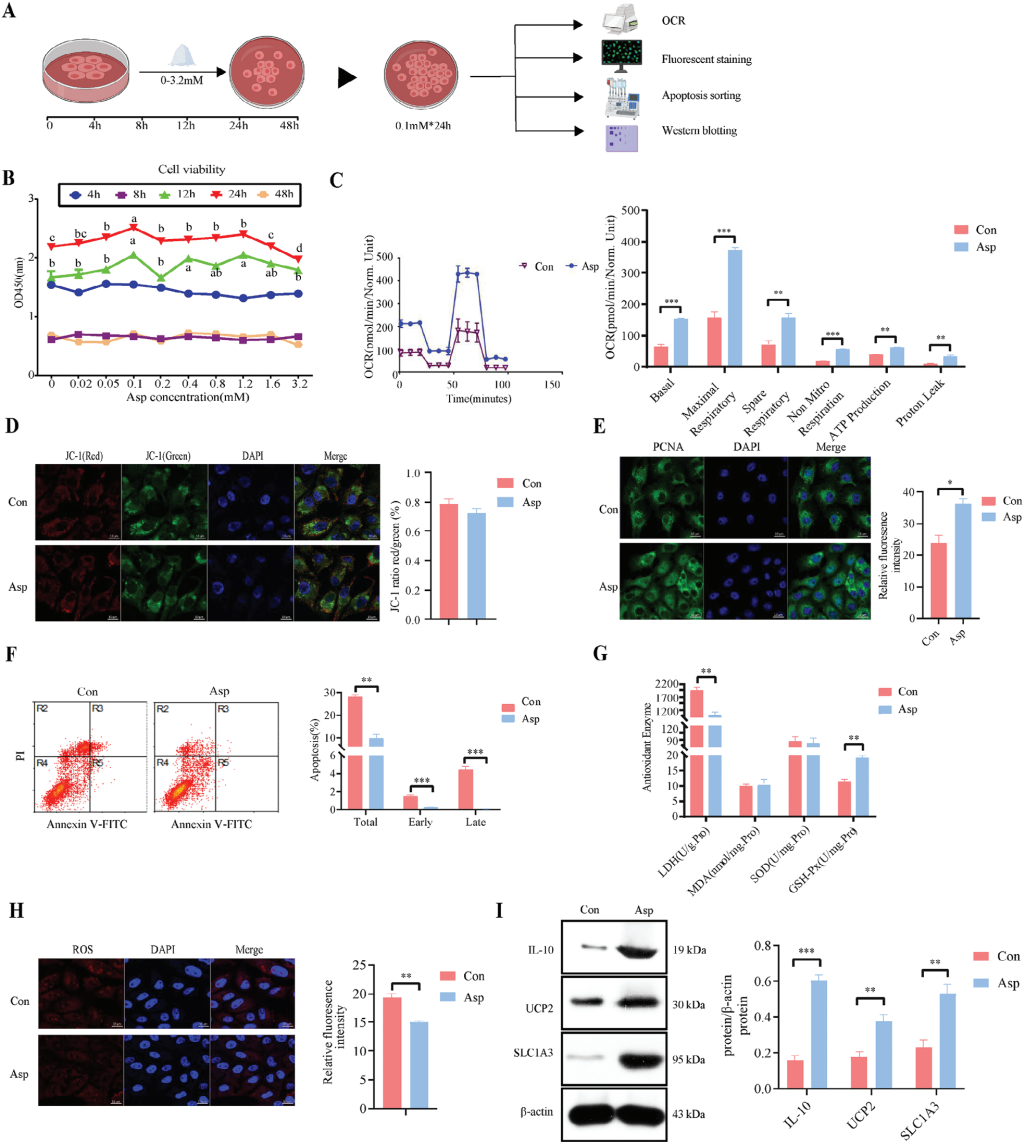
Effects of aspartate on cell proliferation and mitochondrial respiratory metabolism in IPEC‐J2 cells. A) Screening the optimal concentration of Asp added to IPEC‐J2 cells and further testing the efficacy of Asp. B) IPEC‐J2 cells were treated with different concentrations and times to screen the most suitable concentration of Asp. Where the horizontal axis represents the concentration size and the colors represent different treatment times (n = 6 per group). C) Oxygen consumption (OCR) was measured using a hippocampal respiratory metabolism meter (n = 3 per group). D) Mitochondrial membrane potential was detected by staining with JC‐1 dye and observed by confocal microscopy (Scale bar:10 µm): red, aggregates; green, monomers (n = 6 per group). E) Effect of Asp on the expression of proliferative protein PCNA in IPEC‐J2 cells (Scale bar:10 µm, n = 3 per group). F) The total apoptosis rate was detected using the combination of PI and FITC dyes, with necrotic or late apoptotic cells in the R3 region, normal cells in the R4 region, and early apoptotic cells in the R5 region F in the figure (n = 3 per group). G) The activity or concentration of antioxidant paraments (n = 3 per group). H) MitoSOX‐based confocal microscope detection of mitochondrial ROS production (Scale bar:10 µm, n = 5 per group). I) Protein expression of SLC1A1, IL‐10 and UCP2 (n = 6 per group). Statistics: Data are presented as mean ± SEM. Test: Data were analyzed using unpaired two‐tailed Student's *t*‐test, except for the data in panel B, which were analyzed using one‐way ANOVA with Tukey's multiple comparison test. *p < 0.05, **p < 0.01, ***p < 0.001.

Intestinal porcine epithelial cells (IPEC‐J2) are employed for our experiments to evaluate the role of Asp. An optimal increase in IPEC‐J2 cell viability was observed with Asp treatment at a 0.1 mM concentration for 24 h, establishing it as the concentration of choice for subsequent experiments (**Figure** **4**A,B). This treatment also enhanced mitochondrial metabolic functions, including the basal metabolic rate, maximum respiration, non‐mitochondrial respiration, proton leak, residual respiratory capacity, and ATP generation without altering mitochondrial membrane potential (Figure 4C,D). Moreover, Asp upregulated proliferating cell nuclear antigen (PCNA) expression and reduced the overall apoptosis rate of IPEC‐J2 cells (Figure 4E,F). Asp also reduced the generation of mitochondrial ROS, which might be related to increased GSH‐Px activity and decreased LDH content (Figure 4G,H). Western blotting analysis revealed that Asp upregulated IL‐10, uncoupling protein 2 (UCP2), and SLC1A3 protein expression in IPEC‐J2 cells, suggesting it promotes cell proliferation, reduces apoptosis, and improves mitochondrial respiratory metabolism by regulating mitochondrial ROS generation (Figure 4I).

### 2.5 Aspartate Improved Intestinal Flora in Oxidative Stress‐Weaned Piglets

**Figure 5:**
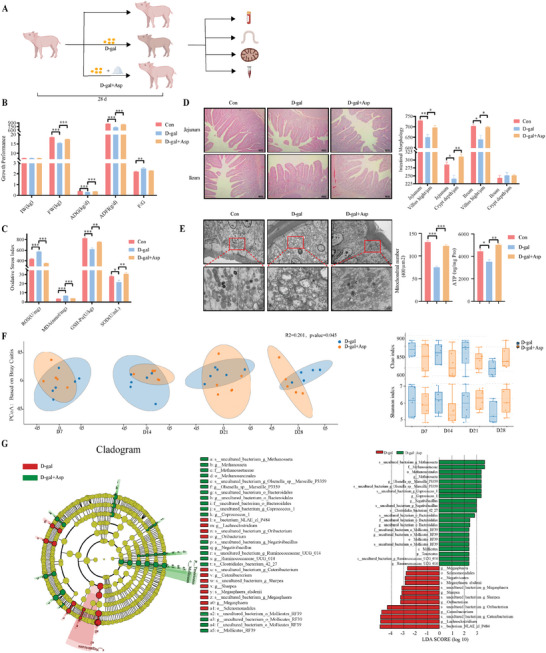
Effects of dietary aspartate supplementation on growth and intestinal microorganisms in oxidative stress piglets. A) The experiment was divided into three groups, and while modeling oxidative stress, Asp was added to the diet to test the antioxidant function of Asp. B) Growth performance (n = 6 per group). C) Serum antioxidant indexes (n = 3 per group). D) Intestinal morphology (Scale bar:100 µm, n = 3 per group). E) Intestinal ultrastructure by transmission electron microscope and intestinal ATP content (Scale bar:1 µm, n = 3 per group). F) The composition and level of intestinal microflora (n = 5 – 6 per group). G) LEfSe is able to look for Biomarkers that are statistically different between groups (n = 6 per group). The circles radiating from inside to outside of the evolutionary branching diagram represent taxonomic levels from phylum to species; each small circle at a different taxonomic level represents a taxon at that level, and the size of the diameter of the circle is proportional to the size of the relative abundance. Statistics: Data are presented as mean ± SEM. Test: Data were analyzed using one‐way ANOVA with Tukey's multiple comparison test except for the data in panel F and G, which were analyzed using unpaired two‐tailed Student's *t*‐test. *p < 0.05, **p < 0.01, ***p < 0.001.

To confirm whether the reversal effects of Asp administration were sufficient to mediate the intestinal flora during long‐term oxidative stress, we examined the growth and gut microbe profiles of oxidative stress piglets (**Figure** **5**A). Under D‐gal‐induced oxidative stress conditions, the ADG and ADFI of weaned piglets fed with 0.5% Asp diet were greater than those in the D‐gal group and did not differ from the control group. Asp also reduced serum ROS and MDA levels, while the activities of GSH‐Px and SOD were increased (Figure 5B,C). Asp enhanced villus height and crypt depth in the jejunum with no significant ideal changes (Figure 5D). Electron microscopy showed that Asp maintained intestinal mitochondrial morphology and increased mitochondrial density, thereby increasing intestinal ATP level and improving energy metabolism under oxidative stress (Figure 5E). These findings indicate that dietary Asp supplementation can improve growth performance and intestinal morphology integrity to alleviate oxidative damage.

**Figure 6:**
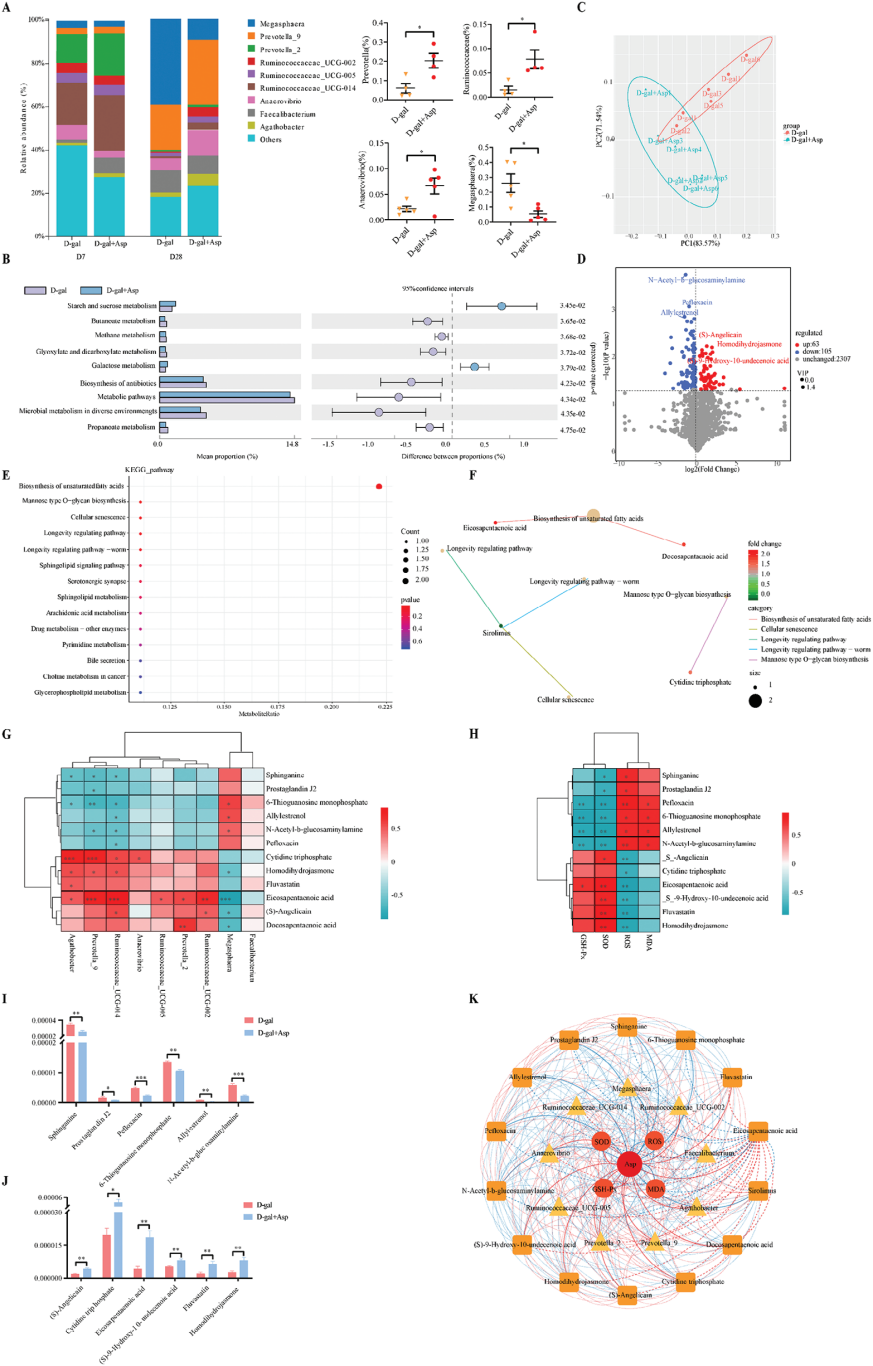
Effect of aspartate on the crosstalk between intestinal microorganisms and metabolites in oxidative stress piglets. A) Species abundance map at the genus level. One color represents one species, and the length of the color block indicates the proportion of relative abundance occupied by the species (only the top ten species at the abundance level are shown, data are presented as mean ± SEM, *p < 0.05, data were analyzed using unpaired two‐tailed Student's *t*‐test except for the *Ruminococcaceae* data, which were analyzed using Mann‐Whitney test, n = 4 – 6 per group). B) Functional prediction analysis of differential microorganisms. C) The principal component analysis demonstrated the overall metabolic differences between the samples in each group and the magnitude of variability between the samples within the groups. D) Metabolite volcano map (VIP: Variable Importance in Projection). E) KEGG enrichment map of differential metabolites. F) Differential metabolite localization map (top five). The yellowish nodes in the figure are pathways, and the small nodes connected to them are specific metabolites annotated to that pathway. G) Pearson correlation coefficients between differential microorganisms and differential metabolites. H) Pearson correlation coefficients between differential metabolites and antioxidant indicators. I, J) Quantification of differential metabolites (unpaired two‐tailed Student's *t*‐test, n = 6 per group, data are presented as mean ± SEM). K) Ternary multi‐dimensional correlation analysis of Asp with microorganisms and metabolites. *p < 0.05, **p < 0.01, ***p < 0.001.

Furthermore, we conducted 16S rRNA sequencing of fecal samples taken at different ages (7, 14, 21, and 28 days) to determine the effect of Asp on the intestinal microbiota profiles in D‐gal‐induced piglets. The Principal Co‐ordinates Analysis indicated a gradual alteration in microbial composition from day 1 to 28, with a significant difference in the Asp‐treated group evident by day 28 compared to the D‐gal group. Species diversity and abundance remained relatively constant (Figure 5F). Linear Discriminant Analysis Effect Size analysis revealed that 37.5% of the differential bacteria were concentrated at the genus level, where significant differences were observed (Figure 5G). We found increases in *Prevotella (2, 9), Ruminococcaceae (UCG002, UCG005, UCG 014)*, and *Anaerovibrio*, and a decrease in *Megasphaera* in the Asp‐supplemented group compared to the D‐gal group (**Figure** **6**A). These alterations in *Ruminococcaceae (UCG002, UCG005)* and *Megasphaera* communities in Asp‐supplemented piglets were opposite to those observed in piglets fed the basal diet under D‐gal treatment. Tax4fun functional prediction showed that the differential microorganisms mainly targeted metabolic pathways, such as sugar metabolism, antibiotic synthesis, and propionate metabolism (Figure 6B). These results suggest that Asp regulates the intestinal microbiota profiles in response to chronic oxidative damage.

### 2.6 The Crosstalk between Aspartate Metabolism and Intestinal Microbiota was Involved in Alleviating Oxidative Damage

Metabolomic analysis of fecal samples was conducted to elucidate the crosstalk between Asp metabolism and differential intestinal microflora under oxidative stress. Principal component analysis revealed significant overall metabolic differences between the D‐gal and D‐gal+Asp groups, with 63 metabolites upregulated and 105 downregulated after Asp treatment (Figure 6C,D). Enrichment analysis via lusterProfiler indicated key pathways, including unsaturated fatty acids biosynthesis, cellular senescence, and mannose‐type O‐glycan biosynthesis. By searching for metabolites within the top five major enrichment pathways (top five), we identified the main differential metabolites, such as EPA, docosapentaenoic acid, sirolimus, and cytidine triphosphate (Figure 6E,F).

Correlation analysis linked differential metabolites to *Prevotella_9, Ruminococcaceae_UCG‐014*, and *Megasphaera*, indicating that the differential metabolites were produced directly by microorganisms. These metabolites, including EPA, S‐angelicain, and cytidine triphosphate, showed positive correlations with antioxidant enzymes GSH‐Px and SOD and negative correlations with ROS and MDA (Figure 6G,H). Absolute quantification indicated Asp treatment increased the levels of S‐angelicaine, cytidine triphosphate, EPA, S‐9‐Hydroxy‐10‐undecenoic acid, fluvastatin, and homodihydrojasmone, aligning with oxidative stress biomarker trends (Figure 6I,J). Asp's comprehensive impact on gut microbiota and metabolites under oxidative stress was demonstrated, highlighting the significant role of different microorganisms in oxidative regulation and their association with metabolite production. EPA emerged as a crucial mediator between microorganisms and antioxidant mechanisms (Figure 6K). These results indicate that Asp alleviated chronic oxidative stress by regulating the crosstalk between intestinal microbiota and Asp‐enhanced production of EPA.

### 2.7 Asp and its Metabolite EPA Alleviated Oxidative Stress in an H_2_O_2_‐Induced Enterocyte Oxidative Stress Model

**Figure 7:**
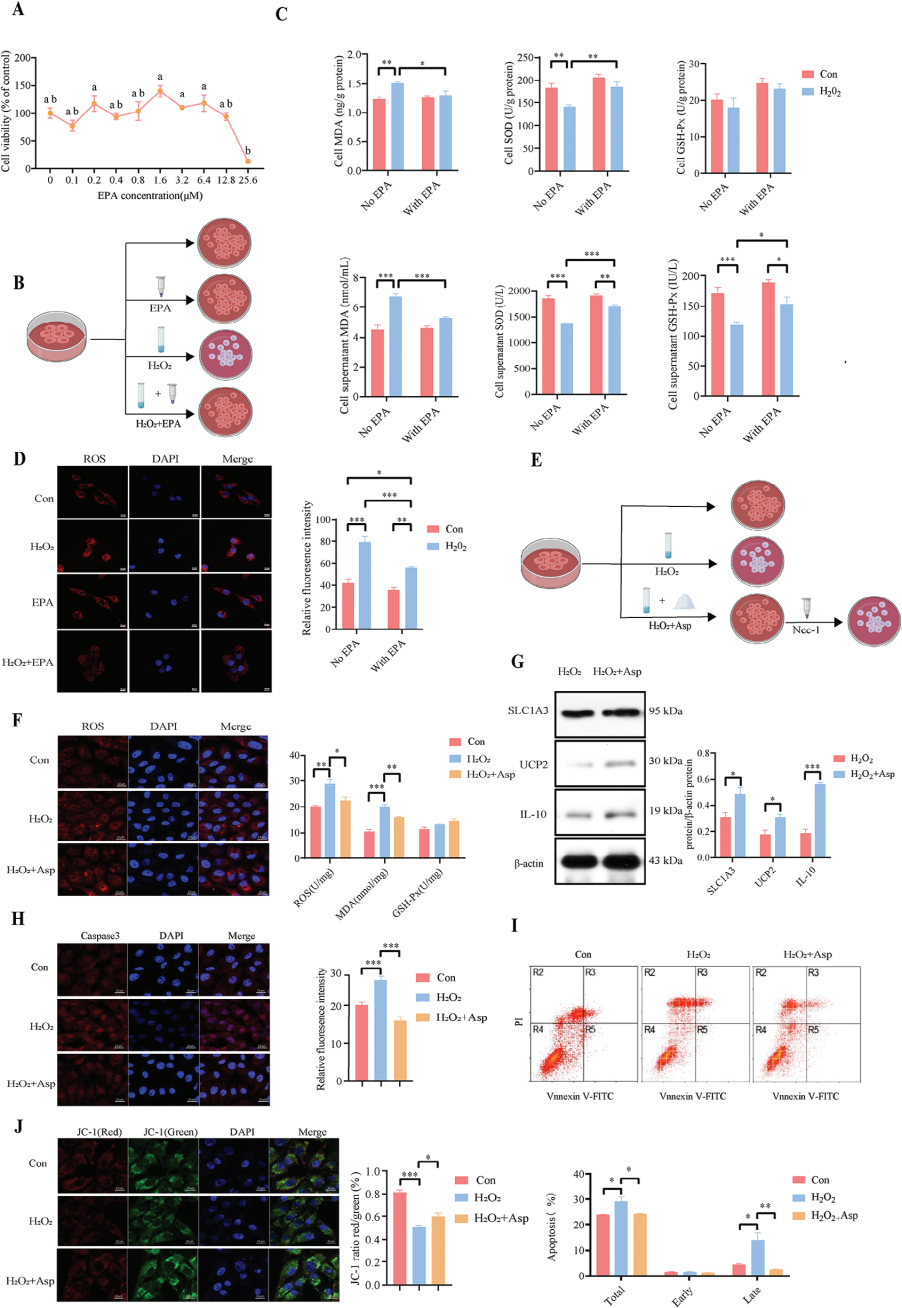
Effects of aspartate on mitochondrial ROS and cell apoptosis in H_2_O_2_‐induced IPEC‐J2 cells. A) Screening the optimal concentration of EPA added to IPEC‐J2 cells and further testing the efficacy of EPA (Kruskal‐Wallis test with Dunn's multiple comparison test, n = 6 per group, data are presented as Mean ± SEM, different letters indicate a difference, *p* < 0.05). B) A diagram of cell treatment. C) The activity or concentration of oxidative stress paraments (two‐way ANOVA with Tukey's multiple comparison test, n = 6 per group, data are presented as Mean ± SEM, *p < 0.05, **p < 0.01, ***p < 0.001). D) Mitochondrial ROS levels in cells treated with EPA and/or H₂O₂. Mito ROS staining (red) and DAPI (blue) indicate mitochondrial ROS and nuclei (Scale bar:10 µm, two‐way ANOVA with Tukey's multiple comparison test, n = 9 per group, data are presented as Mean ± SEM, *p < 0.05, **p < 0.01, ***p < 0.001). E) Experimental strategy diagram of Asp treatment in H_2_O_2_‐induced IPEC‐J2 cells F) Antioxidant parameters in IPEC‐J2 cells (Scale bar:10 µm, unpaired two‐tailed Student's *t*‐test, n = 3 per group, data are presented as Mean ± SEM, *p < 0.05, **p < 0.01, ***p < 0.001). G) The protein expression of SLC1A3, IL‐10, and UCP2 (unpaired two‐tailed Student's *t*‐test, n = 3 per group, data are presented as Mean ± SEM, *p < 0.05, ***p < 0.001). H) Cell apoptotic protein expression (Scale bar:10 µm, one‐way ANOVA with Tukey's multiple comparison, n = 6 per group, data are presented as Mean ± SEM, ***p < 0.001). I) The rate of cell apoptosis (one‐way ANOVA with Tukey's multiple comparison, n = 3 per group, data are presented as Mean ± SEM, *p < 0.05, **p < 0.01). J) Mitochondrial membrane potential changes (Scale bar:10 µm, one‐way ANOVA with Tukey's multiple comparison, n = 6 per group, data are presented as Mean ± SEM, *p < 0.05, ***p < 0.001).

To further investigate the role of EPA as Asp metabolite in alleviating oxidative stress, we initially assessed cell viability at various concentrations of EPA in IEPC‐J2 cells. The results indicated that 1.6 µM EPA produced the highest cell viability, making it the optimal concentration for subsequent experiments (**Figure** **7**A). We then treated cells with H₂O₂ and EPA to examine the impact on oxidative stress markers, showing that H₂O₂ significantly increased MDA level and decreased SOD activities in the cells and supernatants. Additionally, H₂O₂ reduced GSH‐Px activity in the cell supernatants. However, EPA treatment reversed these effects, indicating EPA's protective role against oxidative damage (Figure 7B,C). Moreover, mitochondrial ROS levels, elevated by H₂O₂ treatment, were significantly reduced by EPA, confirming that EPA alleviates oxidative stress (Figure 7D). In line with these findings, Asp treatment decreased mitochondrial ROS and MDA level in IPEC‐J2 cells (Figure 7E,F). Asp treatment also upregulated SLC1A3, IL‐10, and UCP2 protein expression (Figure 7G). Immunofluorescence revealed that H_2_O_2_ increased caspase 3 expression and overall apoptosis, effects that were counteracted by Asp (Figure 7H). Asp also diminished late‐stage apoptosis and enhanced mitochondrial membrane potential in early‐stage stressed IPEC‐J2 cells (Figure 7I,J). These findings indicate that both EPA and Asp effectively mitigate oxidative damage.

### 2.8 Aspartate Mediated the RIP3‐MLKL and RIP1‐Nrf2‐NF‐κB Pathways to Counteract Intestinal Oxidative Damage In Vivo and In Vitro

**Figure 8:**
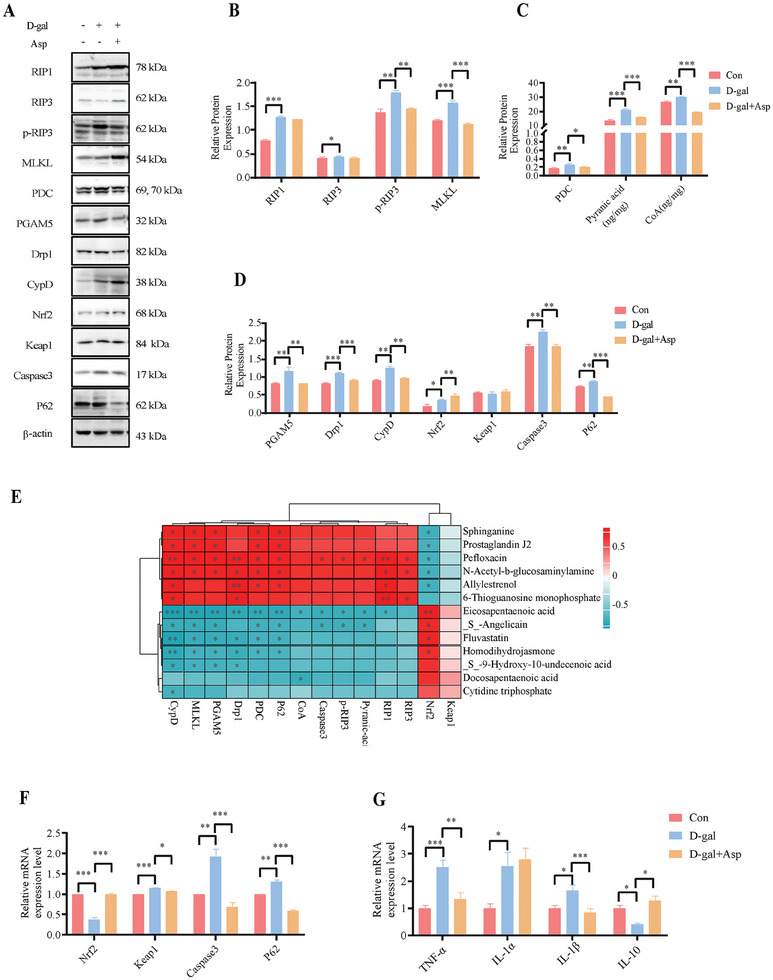
Aspartate metabolism alleviates intestinal oxidative damage by regulating RIP‐dependent mitochondrial function in the piglet model. A) Visualization of western blotting bands after Asp action under oxidative stress conditions. B) Effect of Asp on RIP pathway‐related proteins (one‐way ANOVA with Tukey's multiple comparison, n = 3 per group, data are presented as Mean ± SEM). C) Effect of Asp on pyranic acid and CoA levels affecting mitochondrial respiration in the PDC pathway (one‐way ANOVA with Tukey's multiple comparison, n = 3 per group, data are presented as Mean ± SEM). (D) Effect of Asp on the PGAM pathway affecting mitochondrial function and apoptosis and autophagy proteins (one‐way ANOVA with Tukey's multiple comparison, n = 3 per group, data are presented as Mean ± SEM). E) Heatmap of association analysis of protein expression with intestinal metabolites (Pearson correlation). F) Effect of Asp on relative mRNA expression of antioxidant‐related genes (one‐way ANOVA with Tukey's multiple comparison, n = 3 per group, data are presented as Mean ± SEM). G) The relative mRNA expression of inflammatory cytokines (one‐way ANOVA with Tukey's multiple comparison, n = 6 per group, data are presented as Mean ± SEM). *p < 0.05, **p < 0.01, ***p < 0.01.

Recent reports reported that the catalytic activity of the RIP‐dependent pathway was indispensable in oxidative cell death. This pathway is involved in various cellular signals regulating inflammation, survival, and death.^[^ ^30^ ^]^ Investigating the antioxidant role of Asp, we assessed the RIP‐dependent pathway's expression. The western blotting results indicated that oxidative stress upregulated the protein expression of intestinal RIP1, p‐RIP3, and MLKL, whereas Asp treatment downregulated the protein expression of intestinal p‐RIP3 and MLKL in the D‐gal‐induced piglets, indicating that Asp might counteract intestinal oxidative damage by regulating the RIP signaling pathway (**Figure** **8**A,B). Asp also reduced intestinal PDC protein expression and pyranic acid and acetyl‐coenzyme A levels (Figure 8C), indicating decreased cellular oxygen consumption and ROS production. D‐gal exposure increased PGAM5, Drp1, and CypD protein expression, whereas adding Asp downregulated these proteins (Figure 8D), indicating that Asp could prevent mitochondrial dysfunction and cell necrosis by inhibiting mitochondrial swelling and division. Correlation analysis between RIP pathway proteins and Asp‐related metabolites revealed negative correlations between EPA and RIP1/3 (Figure 8E). These results suggest that Asp reduces cell necroptosis and maintains mitochondrial function by regulating the RIP pathway and through the involvement of its metabolites.

**Figure 9:**
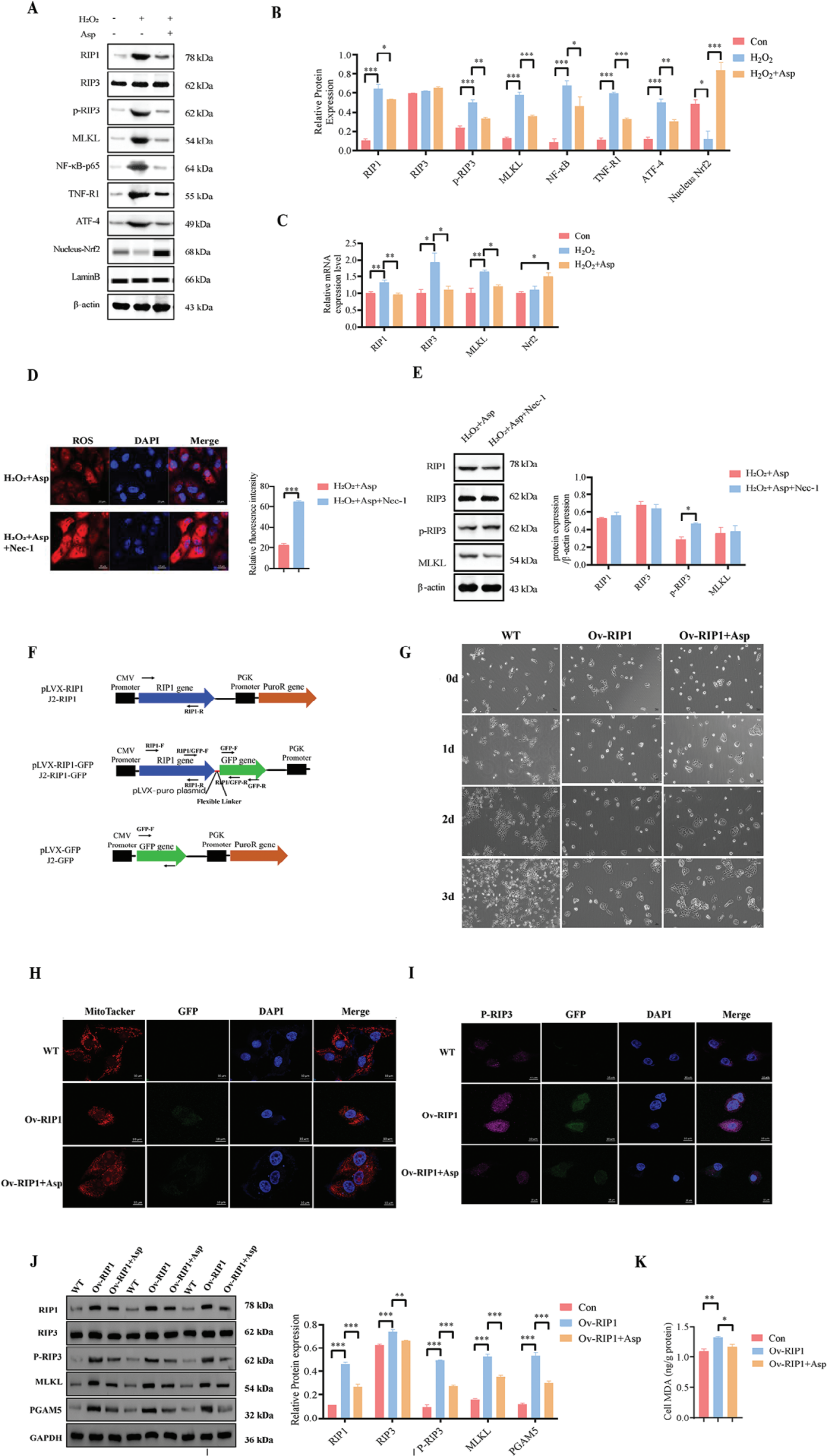
Aspartate eliminates excessive ROS production by mediating RIP pathway IPEC‐J2 cells. A) Visualization of western blot bands after Asp supplementation under oxidative stress conditions in IPEC‐J2 cells. B) Effects of Asp on the expression of related proteins under oxidative stress conditions in IPEC‐J2 cells (one‐way ANOVA with Tukey's multiple comparison, n = 3 per group, data are presented as Mean ± SEM). C) The mRNA expression of related genes (one‐way ANOVA with Tukey's multiple comparison, n = 3 per group, data are presented as Mean ± SEM). D) Changes in ROS content in IPEC‐J2 cells after the addition of Nec‐1, a RIP pathway inhibitor, under conditions where ASP exerts antioxidant effects (Scale bar:100 µm, unpaired two‐tailed Student's *t*‐test, n = 3 per group, data are presented as Mean ± SEM). E) The RIP pathway‐related protein expression (unpaired two‐tailed Student's *t*‐test, n = 3 per group, data are presented as Mean ± SEM). F) Schematic of RIPK and RIPK‐GFP overexpression plasmids. G) Cell morphology of wild‐type (WT) cells, RIP1‐overexpressing (Ov‐RIP1) cells, and Ov‐RIP1 cells cultured with Asp (Scale bar:50 µm). H) Mitochondrial morphology of WT cells, Ov‐RIP1 cells, and Ov‐RIP1 cells treated with Asp. MitoTracker staining (red) highlights the mitochondria, GFP (green) indicates RIP1 overexpression, and DAPI staining (blue) marks the nuclei (Scale bar:10 µm). I) Immunofluorescence analysis of P‐RIP3 expression in WT cells, Ov‐RIP1 cells, and Ov‐RIP1 cells treated with Asp. P‐RIP3 (magenta) indicates phosphorylation of RIP3, GFP (green) marks RIP1 overexpression, and DAPI (blue) stains the nuclei (Scale bar:10 µm). J) Western blot analysis and quantification of RIP1, RIP3, P‐RIP3, MLKL, and PGAM5 protein expression in WT cells, Ov‐RIP1 cells, and Ov‐RIP1 cells treated with Asp (one‐way ANOVA with Tukey's multiple comparison, n = 3 per group, data are presented as Mean ± SEM). (K) Cellular MDA levels in WT cells, Ov‐RIP1 cells, and Ov‐RIP1 cells treated with Asp (Kruskal‐Wallis test with Dunn's multiple comparison test, n = 6 per group, data are presented as Mean ± SEM). *p < 0.05, **p < 0.01, ***p < 0.001.

Oxidative stress, a major factor in intestinal diseases, causes a complex interplay of inflammation, immune response, cell apoptosis, and autophagy.^[^ ^33^, ^34^, ^35^ ^]^ Our research supported these connections, showing that D‐gal increased the mRNA expression of inflammatory factors cytokines (TNF‐α, IL‐α, IL‐β), Keap1, and the protein expression of RIP1, caspase3, and p62 in the intestine (Figure 8A,D,F), while decreasing the Nrf2 and IL‐10 mRNA expression (Figure 8F,G). Asp treatment significantly reduced RIP1, caspase3, p62 protein expression, Keap1 and inflammatory cytokines mRNA expression while restoring Nrf2 protein and IL‐10 mRNA expression. Similar results were obtained in H_2_O_2_‐induced IPEC‐J2 cells with Asp decreasing H_2_O_2_‐induced upregulation of RIP1, NF‐κB‐p65, p‐RIP3, activating transcription factor 4 (ATF4), and TNF‐R1 proteins, while increasing nuclear Nrf2 protein expression (**Figure** **9**A,C). These results indicate that Asp has the ability to alleviate oxidative damage by regulating the activation of the RIP1‐Nrf2‐NF‐κB signaling pathway.

The in vitro experiment further confirmed that H_2_O_2_ exposure significantly upregulated RIP1, p‐RIP3, and MLKL protein expression in IPEC J2 cells. Asp supplementation reduced the levels of p‐RIP3 and MLKL, aligning with in vivo findings (Figure 9A,C). To verify Asp's regulatory effect on the RIP pathway, we used Necrostatin‐1 (Nec‐1) (Figure 7A), an inhibitor of programmed necroptosis, which confirmed that Asp did not decrease mitochondrial ROS level and p‐RIP3 expression during Nec‐1 treatment. Furthermore, Asp had no marked impact on the expression of RIP1, RIP3, or MLKL (Figure 9D,E). To further explore this regulatory effect, RIP1 was overexpressed in IPEC‐J2 cells. RIP1 overexpression inhibited cell proliferation, while Asp supplementation alleviated these adverse effects (Figure 9F,G and Figure S2, Supporting Information). Mitochondrial morphology also improved with Asp treatment, where RIP1 overexpression induced mitochondrial fragmentation and Asp reversed these effects (Figure 9H). Immunofluorescence analysis showed that p‐RIP3 expression was elevated in RIP1‐overexpressing cells, but Asp treatment significantly reduced p‐RIP3 levels (Figure 9I). Western blot analysis confirmed that RIP1 overexpression upregulated RIP1, RIP3, p‐RIP3, MLKL, and PGAM5, while Asp reduced the expression of p‐RIP3, MLKL, and PGAM5 (Figure 9J). MDA levels were elevated in RIP1‐overexpressing cells, but Asp treatment reduced these levels, further supporting Asp's role in reducing oxidative stress (Figure 9K). These results demonstrate that Asp supplementation mitigates oxidative stress and improve mitochondrial function by modulating the RIP pathway, while confirming that the inhibition of the RIP pathway is indispensable for Asp's antioxidant effects.

## 3 Discussion

The gut microbiota and its metabolism are associated with chronic oxidative stress, which typically accelerates imbalances in gut microbial diversity and causes related intestinal diseases.^[^ ^36^, ^37^, ^38^ ^]^ Although there is growing interest in the relationship between oxidative stress and the gut microbiota, it remains unclear whether gut microbiota is mechanistically involved in chronic oxidative stress in a piglet model. Our study found that D‐gal administration induced chronic oxidative stress damage associated with altered gut microbiota and Asp metabolism in piglets. Chronic oxidative stress slowly decreased the species abundance and diversity of microbial populations from day 1 to 28, with a gradual decrease in beneficial bacteria *Ruminococcacea*e (UCG‐002 and UCG‐005). In contrast, harmful bacteria *Megasphaera* increased, suggesting that the damage caused by D‐gal is slow and persistent. *Ruminococcaceae* can convert primary bile acids to secondary bile acids.^[^ ^39^, ^40^ ^]^ *Megasphaera*, a harmful bacterium, is associated with various diseases such as lung cancer, HIV, and bacterial vaginosis, and is usually accompanied by inflammation, indicating a decline in immunity.^[^ ^38^, ^41^, ^42^ ^]^ Thus, these changes in intestinal microbiota are often accompanied by inflammation. Serum ALP, LDH, and LIPC levels often reflect mild inflammation. Some studies have reported that increased serum ALP and LDH levels are related to neuropathic inflammation, which may lead to pain.^[^ ^43^, ^44^ ^]^ Decreased LIPC levels exacerbate oxidative damage, causing systemic inflammation and cardiovascular risk.^[^ ^45^, ^46^ ^]^ This is consistent with our findings, indicating that changes in the intestinal microbiota are a component of chronic oxidative stress and may be a major trigger for inflammation in oxidative damage.

Intestinal microbiota metabolism is closely linked to amino acids, such as Asp, Try, and Gln, which are key driving factors for the anti‐stress effect of digestive Streptococcus.^[^ ^42^, ^47^ ^]^ We found that the metabolites produced by the intestinal microbiota under chronic oxidative stress mainly accumulate in the KEGG pathways that affect respiratory metabolism, protein transport, and amino acid metabolism. The differential metabolic products were mainly related to Asp metabolism and positively correlated with Asp. This is also confirmed by the decrease in free Asp content in the intestinal chyme. The interconnections between changes in the microbiota, altered metabolites, and Asp utilization speculate that chronic oxidative stress may stimulate increased consumption of Asp in the gut, which accelerates energy deprivation and dysfunction. Although piglets are able to synthesize endogenous Asp to primarily meet bodily needs under non‐stressed conditions, reduced synthesis or excessive consumption of Asp in the gut can lead to an inadequate endogenous Asp supply, affecting the diverse gut microbiota.^[^ ^48^, ^49^ ^]^ During oxidative stress, enterocytes produce more ROS and energy consumption increases, resulting in increased utilization of Asp in various key functions, including mitochondrial function, cell proliferation and antioxidant defense.^[^ ^50^ ^]^ Furthermore, endogenous Asp synthesis can vary depending on the health and development of the animal. During weaning, piglets experience rapid growth and physiological challenges, which further limits the body's ability to produce sufficient endogenous Asp.^[^ ^51^ ^]^ Therefore, in order to confirm the higher demand for aspartate during oxidative stress, we chose to study Asp and hypothesized that Asp metabolism is a critical antioxidant node that influences the gut microbiota and metabolites.

Asp supplementation has been linked to enhancing energy metabolism and cell proliferation, observations that align with our studies in piglets. SLC1A1 and SLC1A3 carriers are known to play a crucial role in mitochondrial function and energy metabolism, which is essential for the transport of amino acids. These carriers facilitate the efficient entry of Asp into cells by supporting the energy requirements necessary for its active transport.^[^ ^52^, ^53^ ^]^ The upregulation of transport proteins after adding exogenous Asp facilitates the entry of Asp from the extracellular domain into the intracellular environment and further into the mitochondria, increasing the number of mitochondria and the respiratory metabolism to improve the ATP energy supply. Adequate energy also supports the metabolism and synthesis of amino acids such as Thr, Glu, and Gly from Asp, providing cells and organelles with the necessary amino acids for growth processes and further promoting cell proliferation and immune cell differentiation. PCNA, a marker protein of cell proliferation, plays a crucial role in DNA replication and repair.^[^ ^54^, ^55^ ^]^ UCP2 can prevent mitochondrial Ca^2+^ overload and reduce ROS formation, inhibiting cell apoptosis.^[^ ^56^ ^]^ Under the effect of Asp, the protein expression of PCNA and UCP2 increased and the overall apoptotic rate reduced in IPEC‐J2 cells. This indicates that Asp could promote intestinal cell renewal and differentiation, increasing piglet digestibility and nutrient uptake. Meanwhile, the opposite results of MitoROS and IL‐10 in IPEC‐J2 cells suggest that Asp has antioxidant and immunomodulatory effects. In a non‐oxidative stress state, Asp metabolism mainly promotes growth and development rather than antioxidation.

When weaning piglets experience oxidative stress, Asp plays a significant role in antioxidant functions, mainly by improving intestinal and mitochondrial functions. We observed a significant improvement in intestinal integrity and function in a piglet model of oxidative stress, and this change was related to exogenous Asp addition, further confirming the role of Asp in protecting the intestinal barrier during the progression of chronic oxidative stress. In particularly, we observed that Asp supplementation also led to the simultaneous increase in both villus height and crypt depth, suggesting that Asp promotes cell proliferation. Increased crypt depth is generally linked to enhanced intestinal regeneration, as it indicates a more active proliferative zone where cells rapidly replace damaged ones.^[^ ^57^ ^]^ So this further confirms that Asp is important for supporting both nutrient absorption and intestinal regeneration under conditions of increased metabolic demand or oxidative stress, aligning with previous reports in the literature on similar effects of dietary additives like *Bacillus subtilis* and niacin.^[^ ^57^ ^]^

In actual production, chronic oxidative stress caused an imbalance in the gut microbiota, disordered microbiota also affected the progression of oxidative damage, and there were significant fluctuations in the abundance and diversity of gut microbiota species with a continuous supply of exogenous Asp. Linking the positive feedback between Asp and bacterial metabolism will help solve unexplained antioxidant mechanisms and improve clinical treatment. Regarding the species of bacteria, the abundance of *Prevotella*, *Ruminococcaceae*, and *Anaerovibrio* in the intestine increased by Asp treatment in piglets with chronic oxidative stress, while the content of *Megasphaera* decreased. We speculate that this may be due to the metabolism of the supplemented Asp. Asp not only serves as an energy source, potentially supporting the growth of beneficial bacteria, but it may also influence the gut environment in a manner that discourages the proliferation of pathogenic species. This dual action could lead to an enhanced presence of health‐promoting bacteria, while simultaneously suppressing harmful ones, thus contributing to a more balanced and healthier gut microbiome. Additionally, Asp's involvement in various metabolic pathways, including those related to antioxidant defense, further underscores its potential in maintaining gut health and preventing dysbiosis.

Most amino acids in the gut, including Asp, are derived from the metabolism of gut bacteria, and it is known that *Prevotella* and *Ruminococcaceae* produce Asp and metabolites*. Prevotella* is often associated with the breakdown of carbohydrates and protein fermentation, contributing significantly to the production of amino acids like Asp. *Ruminococcaceae*, another key player in Asp metabolism, is involved in the fermentation of complex plant polysaccharides. The metabolic activities of *Ruminococcaceae* not only contribute to the production of Asp but also to the generation of short‐chain fatty acids which have been shown to have antioxidative properties. Moreover, the metabolism of Asp by these bacteria may influence various aspects of host physiology, including modulation of the gut‐liver axis and the systemic immune response, which are integral in managing oxidative stress. The ability of these bacteria to metabolize Asp and produce beneficial metabolites underlines their potential role in protective mechanisms against oxidative stress‐related damage in the gut. In our study, the metabolic products generated by the gut microbiota under the addition of Asp included EPA, S‐angelicaine, and cytidine triphosphate. Initially, they affect metabolic pathways, such as sugar metabolism, antibiotic synthesis, and propionate metabolism, and are closely related to ROS and antioxidant enzymes (e.g., MDA, GSH‐Px, and SOD). Changes in the abundance of *Prevotella* and *Ruminococcaceae* in the microbiota were strongly and positively correlated with EPA levels. These results suggest that Asp may regulate the increase in *Prevotella* and *Ruminococcaceae* to produce EPA and the antioxidant function of Asp depends on the modulation of gut microbiota. Our findings indicate that EPA treatment in H₂O₂‐induced enterocytes increases antioxidative enzyme activities (SOD and GSH‐Px) and reduces MDA and mitochondrial ROS levels. This suggests that EPA plays a direct role in neutralizing ROS and preventing mitochondrial dysfunction during oxidative stress. These results align with previous reports indicating that EPA plays a critical role in modulating inflammatory responses and oxidative stress in various cellular contexts, including cardiovascular diseases.^[^ ^58^, ^59^ ^]^ Physiological studies have also reported its essential role in improving cell membrane function, cellular respiration, and energy transfer in recombinant *Escherichia coli* containing EPA.^[^ ^60^ ^]^ Therefore, we speculate that Asp might decrease the production of ROS and MDA by altering the EPA generated by the microbiota.

In terms of mitochondrial function, emerging evidence has described the detrimental effects of oxidative stress on mitochondrial metabolism.^[^ ^61^, ^62^ ^]^ For example, stress in lung cancer cells impairs mitochondrial oxidative phosphorylation and accelerates glycolysis.^[^ ^63^ ^]^ ROS induces cellular calcium overload and mitochondrial depolarization in mouse models, triggering cell death.^[^ ^64^ ^]^ It has also been reported that a decrease in the mitochondrial membrane potential caused by stress is a marker of the onset of apoptosis.^[^ ^62^ ^]^ Under normal conditions, mitochondria are shuttle‐shaped and elliptical.^[^ ^10^ ^]^ We found that chronic oxidative stress triggered significant swelling and deformation of mitochondria, accompanied by fission and fusion, drastically reducing the number of mitochondria and ATP production and decreasing energy supply, implying further limitations of antioxidant metabolism and piglet growth. Moreover, Asp administration maintained the morphological structure and number of mitochondria, safeguarded normal mitochondrial function, increased ATP production with a significant decrease in ROS, restored the membrane potential of mitochondria, and decreased the total apoptotic and mortality rates of enterocytes. Asp plays a critical role in eliminating excess mitochondrial ROS and at the systemic level in regulating energy metabolism. This protective mechanism requires linkage to multiple proteins and targets; therefore, we hypothesized that the antioxidant function of Asp relies on a complete and efficient pathway.

Glutamine, a metabolite of Asp, has recently been reported to be involved in RIP3‐mediated cell necroptosis.^[^ ^10^ ^]^ It has also been reported that RIP1 deficiency specifically increases Asp levels and the production of ATP in mice and mammalian cells.^[^ ^10^, ^30^ ^]^ In our study, under oxidative stress conditions, Asp reduced the expression of p‐RIP3 and MLKL in the intestine and further reduced the production of mitochondrial ROS through two pathways: p‐RIP3‐PDC and p‐RIP3‐MLKL. Han's study has revealed that p‐RIP3 promotes cellular respiration and ROS production under TNF stimulation by activating PDC.^[^ ^10^ ^]^ Our results showed that Asp treatment decreased the expression of PDC in the intestine, leading to a reduction of succinate and acetyl‐CoA content, indicating that Asp inhibits the efficiency of ROS production by intestinal cells during aerobic respiration by inhibiting PDC from catalyzing the generation of acetyl‐CoA entering the TCA cycle. Additionally, after being activated by p‐RIP3, MLKL is translocated to the cell membrane. They pump calcium ions, causing an increase in membrane permeability. DAMP molecules such as IL‐1α, IL‐1β, and HMGB1 leak out of the cell, triggering inflammation. At the same time, MLKL can activate Drp1 and CypD in the mitochondria through PGAM5. Drp1 causes ROS accumulation in mitochondria, which subsequently undergoes fission.^[^ ^65^ ^]^ CypD is the main regulatory factor of the mitochondrial permeability transition pore, and depletion or inhibition of the drug can inhibit mitochondrial dysfunction and cell apoptosis induced by NRC‐03.^[^ ^66^ ^]^ In this study, Asp treatment downregulated the protein expression of MLKL, PGAM5, Drp1, and CypD under oxidative stress, confirming the role of Asp in protecting mitochondrial function and cell membrane integrity by mediating the MLKL‐PGAM5 pathway. The increase in mitochondrial content and energy supply confirms that Asp ensures normal metabolism and functional operation of the mitochondria, reducing cell necroptosis and apoptosis. The decrease in inflammatory factors TNF‐α and IL‐1β in enterocytes also suggests that the inflammatory response is alleviated with the reduction of MLKL.

We used the targeted inhibitor Nec‐1 to inhibit the RIP pathway and added Asp. The results showed that ROS production in the mitochondria increased after adding the inhibitor, and the expression of RIP1, RIP3, and MLKL in H_2_O_2_‐induced enterocytes did not change. The effect of Asp failed, and only phosphorylated RIP3 increased, which may be due to the phosphorylation of RIP3 that induces cell apoptosis and necroptosis. The RIP1 overexpression offers important mechanistic insights into Asp's regulatory effects on the RIP signaling pathway. We found that RIP1 overexpression in IPEC‐J2 cells accelerated cell necroptosis and mitochondrial fragmentation, while Asp supplementation effectively alleviated these effects by improving cell viability and restoring mitochondrial morphology, suggesting that it plays a crucial role in maintaining mitochondrial dynamics and preventing mitochondrial fission to defend cell necroptosis. Furthermore, the decreased expression of p‐RIP3, MLKL, PGAM5, and Drp1 proteins and MDA content after Asp treatment in RIP1‐overexpression cells provides stronger evidence that Asp can directly interferes with the necroptotic signaling cascade and then retard mitochondrial dysfunction and ROS accumulation to inhibit the programmed cell death and oxidative stress response. These finding collectively highlight that the protective mechanism of Asp in oxidative stress‐induced intestinal damage may be mediated through its dual action of modulating both mitochondrial function and the **RIP1‐RIP3‐MLKL** axis pathway.

A previous study reported that a decrease in RIP1 promotes the transfer of Nrf2 from the cytoplasm to the nucleus in liver cells, thereby inhibiting cell death.^[^ ^67^ ^]^ The interaction between NF‐κB and RIP1 has also been shown to control the onset of early cell death,^[^ ^68^ ^]^ such as in tumor cells, where the activation of RIP1 can directly activate NF‐κB, thereby inducing inflammation and programmed cell death.^[^ ^69^ ^]^ The results of in vivo and in vitro experiments showed that the increase in RIP1 expression inhibited the antioxidant function of Nrf2 in oxidative stress enterocytes, and the activation of NF‐κBp65 increased the secretion of inflammatory factors, exacerbating the inflammatory and oxidative stress responses. The expression of caspase3 and p62 was also increased, suggesting that oxidative stress promotes cell apoptosis and autophagy. It has been reported that the reduction of p62 gene expression can alleviate autophagy in Alzheimer's disease,^[^ ^70^ ^]^ and the mononuclear cell motility inhibitory factor can increase cell survival rate and reduce cell apoptosis by inhibiting the expression of caspase 3 in myocardial cells.^[^ ^66^ ^]^ Consistent with our results, administration of Asp to oxidative stress enterocytes effectively reversed the expression of RIP1, p62, caspase3, and NF‐κBp65 and the levels of inflammatory factors. Only nuclear Nrf2 maintained a high level, possibly due to its endogenous immune function and the influence of Asp. These findings indicate that Asp supplementation may be important for treating oxidative stress. The role of the RIP1‐Nrf2‐NFκB pathway in modulating antioxidant functions has not been reported within the context of Asp metabolism, which is worthy of further study. Additionally, Asp metabolites like EPA are involved in regulating RIP signaling and have a strong correlation, suggests that Asp is directly involved or in the form of metabolites that dynamically mediate the gut microbiota and RIP‐dependent pathway to improve mitochondrial function and inhibit ROS production. The antioxidant function of Asp exists in various forms but is interconnected.

## 4 Conclusion

Collectively, our results demonstrate that protection by Asp is a multifactorial process that synergistically affects piglet growth and antioxidative stress. We provide a multilevel comprehensive picture of the interplay between Asp metabolism and the gut microbiota and reveal that Asp alleviates oxidative stress by mediating RIP‐dependent mitochondrial function. These findings imply a complex and critical connection between amino acid metabolism and the gut microbial functions involved in oxidative stress, which may help design future intervention strategies against chronic oxidative stress‐related diseases. Furthermore, they highlight Asp's potential in therapeutic and preventive strategies for gastrointestinal disorders linked to oxidative stress, emphasizing the need for dietary Asp in maintaining intestinal health. Understanding these mechanisms opens new avenues for targeted therapies against chronic oxidative stress‐related diseases. The clinical significance of these findings, particularly in human health, warrants further investigation to validate Asp's role as a vital component in disease prevention and management strategies.

## 5 Experimental Section

### Animals and Samples

All animal studies and protocols complied with the procedures approved by the Animal Ethics Committee of the Institute of Subtropical Agriculture, Chinese Academy of Sciences (2 013 020; Changsha, China). A batch experiment was conducted using healthy piglets (Duroc × Landrace × Large Yorkshire) weaned at 28 days with an average body weight (BW) of 9.74 ± 0.15 kg. In the first selection, 20 piglets were randomly divided into two groups, with 10 piglets in each group. The control group was fed the basal feed, while the other group was fed 10 g kg^−1^ BW of D‐galactose (D‐gal) to establish a chronic oxidative stress model.^[^ ^32^ ^]^ In the second selection step, 40 piglets were randomly divided into four groups (n = 10 per group). They were fed with basic feed supplemented with 0%, 0.25%, 0.5%, or 1.0% of aspartate (Asp). In the third selection, 30 piglets were randomly divided into three groups (n = 10 per group). They were fed with basic feed, basic feed supplemented with 10 g kg^−1^ BW of D‐gal, or basic feed supplemented with 10 g kg^−1^ of D‐gal and 0.5% of aspartate (D‐gal+Asp). The feed composition and nutritional levels met the nutritional requirements for pigs weighing 5–10 kg (Table S1, Supporting Information). All animals had ad libitum access to feed and water and were euthanized when necessary to alleviate suffering. No feed was provided the night before slaughter. Fecal, blood, and intestinal samples were collected from these weaned piglets, immediately frozen on dry ice, and stored at −80 °C.^[^ ^71^, ^72^ ^]^

### IPEC‐J2 Cell Culture and Treatment

Intestinal porcine epithelial cells (IPEC‐J2) were maintained in DMEM‐H (Thermo Scientific, USA) supplemented with 10% fetal bovine serum (FBS; Hyclone, USA), 5 mM L‐glutamine, 100 U mL^−1^ penicillin, and 100 µg mL^−1^ streptomycin. Cells were cultured in uncoated plastic flasks (either 25 or 100 mm^2^), 6‐well, 24‐well, or 96‐well plates, adjusting for the appropriate cell density. Oxidative stress was induced with 200 µM H_2_O_2_ (Sigma‐Aldrich, USA) for 6 h, while L‐aspartate (Sigma‐Aldrich; ≥99.0% purity) and EPA (Sigma‐Aldrich; ≥98.5% purity) treatment varied in dosage and duration to assess oxidative stress mitigation. Cell viability was assessed under different Asp or EPA concentrations, with or without H_2_O_2_, for set durations. For analysis, cells at ≈60–70% confluence were switched to FBS‐free media with Asp or EPA (Asp: 0 or 0.1 mM, EPA: 0 or 1.6 µM) for 18 h. Post‐incubation, cells were washed with PBS (Hyclone, USA) and exposed to 200 µM H_2_O_2_ with Asp or EPA (Asp: 0 or 0.1 mM, EPA: 0 or 1.6 µM) for another 6 h. Both culture media and cells were then harvested for analysis.

### Measurement of Physiological and Biochemical Assays

Malondialdehyde was determined using the Thiobarbituric acid reactive substances method.^[^ ^73^ ^]^ Cytochrome *c* assay was used to measure SOD activity.^[^ ^74^ ^]^ GSH‐Px activity was calculated according to an established procedure using H_2_O_2_ as substrate.^[^ ^74^ ^]^ ATP content was determined by the firefly luciferin‐luciferase system.^[^ ^75^ ^]^ ROS assays were measured using ELISA kits (Jiangsu Meimian Industrial Co., Ltd, Jiangsu, China) and performed as previously reported.^[^ ^10^ ^]^ Samples for amino acid analysis were determined using an automatic amino acid analyzer (L‐8900; Hitachi Global Inc., Hitachi, Tokyo, Japan).^[^ ^76^ ^]^ Serum levels of ALP, LDH, and LIPC were measured using a Cobas c311 automatic biochemical analyzer (Roche, CH, SE). The detection steps followed the instructions of the reagent kit (Cobas c311, Roche, CH, SE).^[^ ^3^ ^]^

### Histopathological Examination

The intestinal morphology was defined as previously described.^[^ ^3^ ^]^ Villus height and crypt depth were measured on tissue sections using a light microscope with ImageJ software version 1.51 (National Institutes of Health, Bethesda, MD). For ultrastructural analysis, tissues were fixed in 3% glutaraldehyde, processed, and sectioned. Sections were stained with uranium acetate (GZ02616) for 10–15 min and with lead citrate for 1–2 min, then examined under a JEM‐1400PLUS (JEOL, Tokyo, Japan) transmission electron microscope. After electron microscopy, photographs were taken at a magnification of 600×for observation and statistical analysis of mitochondrial morphology and quantity.

### Analysis of Blood T‐Cell Using Flow Cytometry

Blood samples (2 mL) were collected from the anterior vena cava of each fasting pig and placed in 10 mL heparinized tubes. After mixing and resting for 5–10 min, 2 mL of lymphocyte separation medium was added, and samples were centrifuged at 2000 rpm for 10 min. The white blood cell layer was carefully extracted and transferred to fresh tubes with separation medium, then centrifuged at 3000 rpm for 10 min. This step was repeated twice to remove residual red blood cells. Anti‐CD3^+^, Anti‐CD4^+^, and Anti‐CD8^+^ monoclonal antibodies were diluted 1:50, and 100 µL was added to each sample, followed by incubation at 37 °C for 20–30 min. Samples were washed three times with PBS, then incubated with fluorescein isothiocyanate (FITC)‐labeled secondary antibody (mouse anti‐pig IgG) for 20 min at 37 °C. After a final three PBS washes, samples were analyzed by flow cytometry.

### Quantitative Reverse‐Transcription PCR

Total RNA was extracted from tissue samples using TRIzol Reagent. The mRNA expression levels of selected genes (Table S2, Supporting Information) were quantitatively evaluated. The analyses were performed on an Applied Biosystems 9100 Real‐Time PCR System, employing SYBR Green Supermix to detect amplification products.^[^ ^77^ ^]^ The relative expression of each target mRNA was normalized to β‐actin.

### Western Blotting Analysis

Protein extracts were obtained from homogenized, flash‐frozen tissue using RIPA buffer fortified with protease and phosphatase inhibitors to ensure stability. The detailed process was described in the previous report.^[^ ^3^, ^78^ ^]^ Western blotting was conducted using a range of primary antibodies, detailed in Table S3, Supporting Information.

### Cell Apoptosis

Apoptosis in IPEC‐J2 cells was assessed via dual staining with Annexin V‐FITC and propidium iodide (PI), utilizing flow cytometry kits designed for apoptosis detection. In this process, cells were collected and stained using apoptosis detection reagents (BD556547, Beckman Coulter, USA) following the protocol provided by the manufacturer. This staining discriminates between viable, early apoptotic, and late apoptotic or necrotic cells. Flow cytometric analysis was then performed (Beckman Coulter, USA), allowing for the precise enumeration and categorization of cells in each stage of apoptosis. The proportion of apoptotic cells was then calculated, giving a detailed quantitative representation of the apoptotic status within the cell population under investigation.

### Mitochondrial ROS, Morphology and Membrane Potential Determination

Sterilized coverslips were prepared in six‐well plates for cell culture, followed by 24 h of incubation. Mitochondrial ROS levels were assessed by adding 5 µM MitoSOX (1 mL, Invitrogen, California, USA) to each well, incubating the cells at 37 °C in darkness for 10 min, and rinsing with PBS. DAPI (20 µL, Wellbio, Shanghai, China) was subsequently added for nuclear staining for 5 min in the dark. Mitochondrial morphology was evaluated by incubating cells with 250 µM MitoTracker (1 mL, Invitrogen, California, USA) at 37 °C for 25 min, followed by PBS rinsing and DAPI staining. Mitochondrial membrane potential was measured using 10 µg mL^−1^ JC‐1 (1 mL, Invitrogen, California, USA), with incubation for 30 min at 37 °C in the dark and subsequent PBS washing. Imaging for all assays was performed using a Zeiss LSM880 confocal microscope (Carl Zeiss GmbH, Jena, Germany) to capture fluorescence and ensure accurate analysis of mitochondrial status under experimental conditions.

### Mitochondrial Respiration Metabolism

Cells (100 µL) were seeded into the hippocampal respiratory metabolic plate (Zeiss, Changsha, China). After a 4‐h attachment period, 150 µL of DMEM was introduced. On the day before the assay, 1 mL of buffer was added to each well, and the plate was then incubated overnight at 37 °C in a carbon dioxide‐free environment. The working solution and the mother liquor were prepared and added as per the provided instruction manual, and the measurements were subsequently taken using a hippocampal respiration and metabolism meter. Finally, the cell plate was removed, and the total cell protein concentration was determined by the BCA assay kit, which was used to correct the baseline oxygen consumption (OCR).

### Cellular Immunofluorescence

Cells were accessed in a six‐well plate and cultured for 24 h. After rinsing with PBS, cells were fixed by adding 4% paraformaldehyde (Gibco, USA) and rinsed three times with PBS after 15–20 min. The fixed cells were permeabilized and blocked using a solution containing 0.5% Triton X‐100 (Thermo Scientific, USA) and 2% BSA (Thermo Scientific, USA) for 30min‐1h. After removing the blocking solution, cells were incubated with a primary antibody (diluted 1:200) at 4 °C overnight, away from light exposure. The cells were then washed with PBS before adding a secondary antibody (diluted 1:500) and incubating for an additional 1 h at room temperature, ensuring they were shielded from light. Following a PBS wash, the cells were mounted on slides and placed inside an immunohistochemistry dark chamber. Each slide was treated with 20 µL of the nuclear stain DAPI. The coverslips from the six‐well plate were carefully inverted and placed over the stained cells. After protecting from light for 5 min, the slides were examined, and images were captured using a fluorescence microscope.

### 16S rRNA Gene Sequencing and Analysis of Fecal Microbiota

Total DNA was isolated and sequenced on the Illumina HiSeq sequencing platform. This work designed primers targeting conserved regions with appended sequencing adaptors. Following PCR amplification, this work purified, measured, and equilibrated the products to construct sequencing libraries, which, after stringent quality checks, were sequenced using the Illumina HiSeq 2500 system. The high‐throughput sequencing produced raw image files that were transformed into sequences captured in the FASTQ format, encompassing both nucleotide sequences and their quality scores. Subsequent bioinformatic processing included refining reads through filtering, clustering, or noise reduction, annotating species, and quantifying their abundance. This work delved deeper into the compositional differences between samples by examining alpha and beta diversity, pinpointing significant species disparities, and conducting correlation assessments and functional predictions to infer potential metabolic pathways.

### Metabolome Analysis of Feces

Before analysis, the data were normalized using the total peak area normalization method, which involves dividing each metabolite in each sample by the total peak area of that sample, resulting in quantified information for the pooled metabolites. Orthogonal projections to latent structure discriminant analysis (OPLS‐DA) were used for the analysis. More reliable information on intergroup differences and correlations with the experimental group was obtained by filtering out the orthogonal variables unrelated to the categorical variables in the metabolites. The R package ropls (version 3.3.2) were used for the OPLS‐DA model calculations. For biological replicates, a combination of fold change, *t*‐test *p*‐value, and the OPLS‐DA model's VIP value was used to select differential metabolites, with the criteria of FC>1, *p*‐value <0.05, and VIP>1. Enrichment analysis of differential metabolite Kyoto Encyclopedia of Genes and Genomes annotations was performed using the hypergeometric test method with ClusterProfiler.

### Plasmid Constructs and Transfection for RIP1 Overexpression

For the overexpression of RIP1 in IPEC‐J2 cells, this work designed and used three distinct plasmid constructs: pLVX‐RIP1, pLVX‐RIP1‐GFP, and pLVX‐GFP (control). Transfections were performed using Lipofectamine 3000 (Thermo Scientific, USA), following the manufacturer's instructions. After 48 h, transfected cells were selected with puromycin (2 µg mL^−1^) to ensure stable integration of the plasmids. The efficiency of overexpression was confirmed by both Western blotting and fluorescence microscopy for GFP expression.

### Statistical Analysis

All statistical analyses were conducted using GraphPad Prism (version 9.0). Outliers were removed based on a ROUT outlier test (Q = 5%). For comparisons between two groups, this work used an unpaired two‐tailed Student's *t*‐test, while for multiple group comparisons, either one‐way ANOVA or two‐way ANOVA was employed, followed by Tukey's or Dunnett's multiple comparison tests, depending on the dataset. In cases of unequal variances, Welch's correction was applied. Pearson correlation analysis was used to assess relationships between variables, and the Kruskal‐Wallis test with Dunn's multiple comparison test and Mann‐Whitney test was employed for non‐parametric data (e.g., Figure 6A and 7A). Data were presented as mean ± standard error of the mean (SEM). A *p*‐value of less than 0.05 was considered statistically significant. The number of replicates (n) is indicated in the figures or figure legends, where applicable. Detailed statistical methods are provided in the figure legends or within the main text.

## Conflict of Interest

The authors declare no conflict of interest.

## Author Contributions

S.J., J.W., and C.W. contributed equally to this work. S.J., J.W. and C.W. performed most of the experiments. Y.H., L.H. and H.Z. assisted with animal experiments. Y.T., D.L., Z.W., Y.F., H.C., X.H., G.Y., and J.Q. provided help for some experiments. C.P. assisted with the data analysis. S.J., and J.W. analyzed data and wrote the manuscript. T.L., Y.Y., and L.H. supervised this research and edited the paper. All authors provided feedback on the writing.

## Supporting information



Supporting information

## Data Availability

The data that support the findings of this study are available from the corresponding author upon reasonable request.
