# Supplementary material for: Aspartate Metabolism‐Driven Gut Microbiota Dynamics and RIP‐Dependent Mitochondrial Function Counteract Oxidative Stress
Source: Adv Sci (Weinh). 2025 Jan 28;12(11):2404697. doi: 10.1002/advs.202404697 (PMC11923965; doi:10.1002/advs.202404697)
Supplement: Supplementary file 1 — Supporting information [file ADVS-12-2404697-s001.docx]

Supporting Information

**Title:** **Aspartate Metabolism-Driven Gut Microbiota Dynamics and RIP-Dependent Mitochondrial Function Counteract Oxidative Stress**

Shunshun Jin, Jian Wu, Chenyu Wang, Yiwen He, Yulong Tang, Le Huang, Hui Zhou, Di Liu, Ziping Wu, Yanzhong Feng, Heshu Chen, Xinmiao He, Guan Yang, Can Peng, Jiazhang Qiu, Tiejun Li*, Yulong Yin*, Liuqin He*

**Table S1 Basic diet formula and nutrient level of weaner piglets (air dry basis，%)**

| Items | Content | Nutrient levels^2^ |  |
| --- | --- | --- | --- |
| Ingredients |  | CP | 17.03 |
| Soybean meal | 17.80 | DE/(MJ/kg) | 14.55 |
| Corn | 62.50 | Lys | 1.31 |
| Bean oil | 3.00 | Thr | 0.73 |
| Powder | 0.50 | Trp | 0.20 |
| NaCl | 0.30 | Ca | 0.70 |
| Wheat bran | 0.30 | TP | 0.22 |
| Items | Content | Asp | 1.40 |
| Fish meal | 6.00 |  |  |
| Dried whey | 2.63 |  |  |
| Glucose | 3.00 |  |  |
| Lys | 0.80 |  |  |
| Met | 0.47 |  |  |
| Thr | 0.40 |  |  |
| Trp | 0.10 |  |  |
| CaHPO_4_ | 1.20 |  |  |
| Premix1 | 1.00 |  |  |
| Total | 100.00 |  |  |

1）Premix was provided per kg of diet: VA 24 375 IU，VB_1_ 7.5 mg，VB_2_ 18.75 μg， VB_6_ 9 mg，VB_12_ 70 mg，VD_3_ 7000 IU，VE 60 IU，VK_3_ 7.5 mg，choline（VB4，50%）1000mg，Co (as cobalt chloride) 5 mg，Zn 85 mg，Mn 4 mg，Fe 90 mg，Cu 5 mg，Se 0.2 mg，I 0.15 mg。

2）Crude protein was measured value, the rest were calculated value

**Table S2 Primers used for quantitative reverse transcription-PCR**

| Genes | Full name | Serial number | Primer sequences (5’-3’) | Product length /bp |
| --- | --- | --- | --- | --- |
| β-actin | β-actin | XM_003124280.3 | F: CTGCGGCATCCACGAAACT  R: AGGGCCGTGATCTCCTTCTG | 147 |
| TNF-α | Tumor Necrosis Factor-α | NM_214022.1 | F: CCACGTTGTAGCCAATGTCA  R: CAGCAAAGTCCAGATAGTCG | 395 |
| IL-1β | Interleukin-1β | XM_021085847.1 | F: GCTAACTACGGTGACAACAA  R: TCTTCATCGGCTTCTCCACT | 196 |
| IL-6 | Interleukin-6 | NM_001252429.1 | F: CAAAGCCACCACCCCTAAC  R: TCGTTCTGTGACTGCAGCTT | 66 |
| IL-1α | Interleukin-1α | XM_021081650.1 | F: TACTTTGCTGAGCGGGTGAC  R: GAAGGTGTGACTCTCGGCTC | 195 |
| IL-10 | [Interleukin-10](http://www.baidu.com/link?url=TCV3gaFHbzLNRnQ-RGawMilBDq2mNosQsUOJUbd_c2BfbEMTu9SaJDL9VANzzza7BxC7RBpop-uYhfzsKFy9wa) | XM_021081648.1 | F: CGCTGCAACAAGAGGGTCTA  R: ATTCCGTAGCAGGGCTTGTG | 161 |
| SLC1A1 | Solute carrier family 1 member 1 | XM_017321385.2 | F: GCACAGTCAAGGCCGAGAAT  R: GCCTTCTCCATGGTGGTGAA | 151 |
| SLC1A3 | Solute carrier family 1 member 3 | XM_021172037.2 | F: GGTTCGAGCCCAATTTTACA  R: CCCACCAGGAACTTCTCAAA | 199 |
| RIP1 | Receptor-interacting protein kinase 1 | NM_001367995.1 | F: CTCCATGCACGAATTCTCAG  R: ACGTCAGTTTTGCCTCATTG | 117 |
| RIP3 | Receptor-interacting protein kinase-3 | XM_021209691.2 | F: CCACTGCAGGACCTCATTTT  R: CACCTTTGCCCAAGTCATCT | 73 |
| PDC | Pyruvate dehydrogenase complex | XM_003130382.4 | F:ACAGGCCACACATACAGGACC  R:TGTCCTTCTTGCTAGGTGGGA | 101 |
| MLKL | Mixed-lineage kinase domain-like | NM_214131.1 | F: GGATTGAGACGGACAGTGGG  R:CCGTCCTTTGAATTTCGCCA | 124 |
| Nrf2 | NF-E2-related factor 2 | NM_006164.5 | F: GAAAGCCCAGTCTTCATTGC  R:TTGGAACCGTGCTAGTCTCA | 190 |
| Keap1 | Recombinant Kelch Like ECH Associated Protein 1 | XM_003123639.4 | F: GAACGTAGGGGAGAGTGTGG  R:CTTGCTAGGGTCGGAAGAGC | 113 |
| p62 | Ubiquitin binding protein p62 antibody | XM_030255494.1 | F: CGAGACCTGGGGTTCTTTGG  R:TATGGTGCGCATAGTCCATCA | 142 |
| Caspase 3 | Cysteine-aspartic acid protease | NM 214131.1 | F AGCATCCACATCTGTACCA  R CCGGAATGGCATGTCGAT | 38 |

**Table S3 The information of primary antibodies**

| Items | Full name | Serial number | Dilution rate | Source |
| --- | --- | --- | --- | --- |
| Marker | Marker | 26617 | 1：1000 | Thermo |
| Nrf2 | NF-E2-related factor 2 | 16396-1-AP | 1：1000 | Proteintech |
| p62 | Ubiquitin binding protein P62 antibody | 18420-1-AP | 1：1000 | Proteintech |
| CypD | Recombinant Cyclophilin D | 67632-1-Ig | 1：1000 | Proteintech |
| PDC | Pyruvate dehydrogenase complex | 13426-1-AP | 1：1000 | Proteintech |
| Caspase3 | Cysteinyl aspartate specific proteinase 3 | ab13847 | 1：1000 | Abcam |
| ATF4 | Recombinant activating transcription factor 4 | ab131607 | 1：1000 | Abcam |
| Keap1 | Recombinant Kelch Like ECH Associated Protein 1 | ab218815 | 1：1000 | Abcam |
| PGAM5 | Phosphoglycerate mutase family 5 | ab131552 | 1：1000 | Abcam |
| Drp1 | Dynamin-related protein 1 | ab154879 | 1：1000 | Abcam |
| NF-κB-p65 | Transcription factor p65 | 3033s | 1：1000 | CST |
| MLKL | Mixed-lineage kinase domain-like | 37705s | 1：1000 | CST |
| TNF-R1 | Tumor Necrosis Factor-receptor1 | 3736s | 1：1000 | CST |
| IL-10 | Interleukin 10 | ab193811 | 1：1000 | Abcam |
| UCP2 | Mitochondrial uncoupling protein 2 | ab97931 | 1:1000 | Abcam |
| SLC1A3 | Solute carrier family 1 member 3 | 20785-1-AP | 1:1000 | Proteintech |
| RIP1 | Receptor-interacting protein kinase 1 | 3493s | 1:1000 | CST |
| RIP3 | Receptor-interacting protein kinase-3 | 95702s | 1:1000 | CST |
| p-RIP3 | Phosphorylation of receptor-interacting protein kinase-3 | 93654s | 1:1000 | CST |
| β-actin | Beta actin | 66009-1-Ig | 1:5000 | Proteintech |
| Lamin B1 | Lamin B1 | 9087 | 1:1000 | CST |


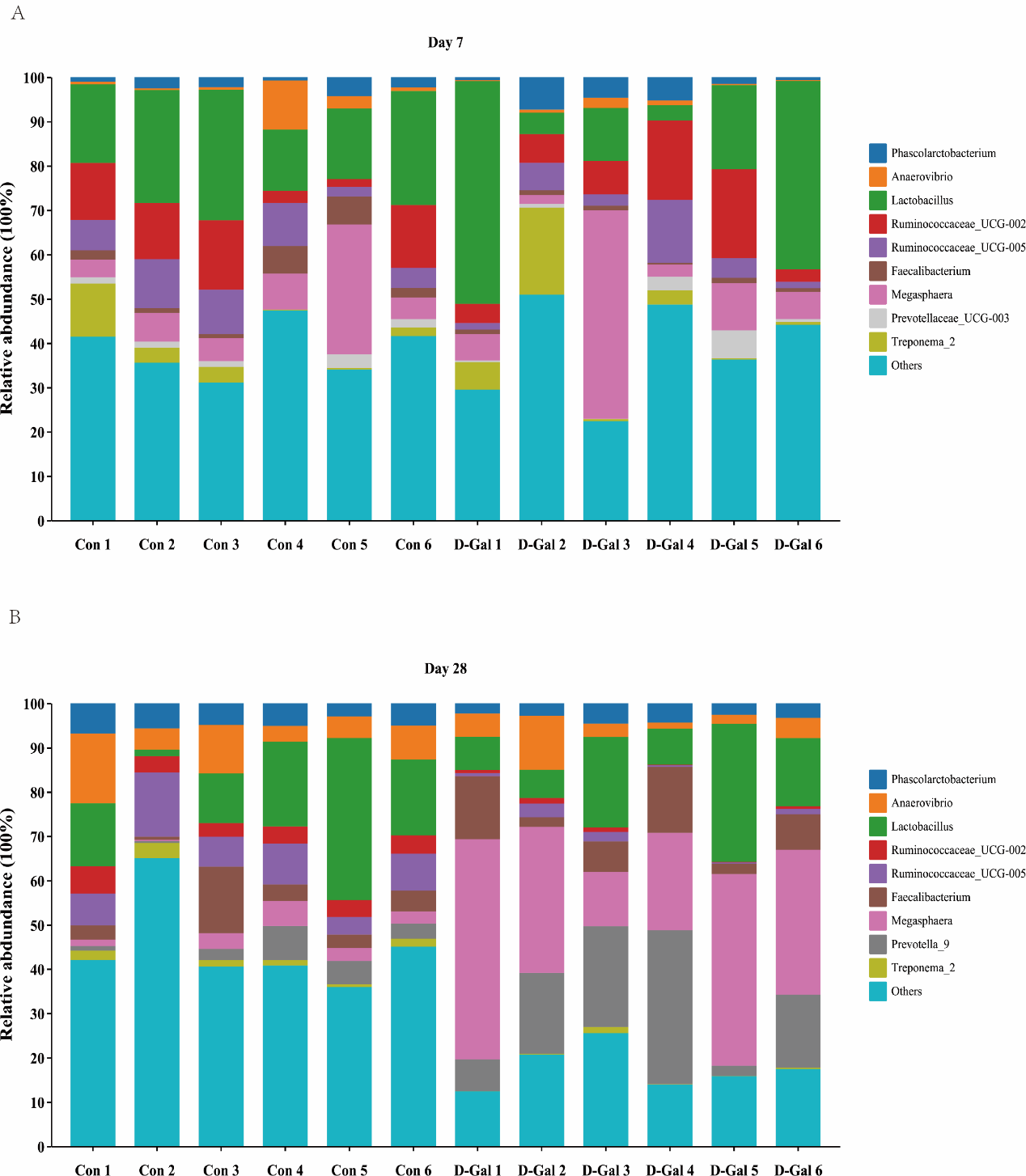


Figure S1. Species abundance map at the genus level in oxidative stress piglet model. One color represents one species, and the length of the color block indicates the proportion of relative abundance occupied by the species (only the top ten species at the abundance level are shown). n = 6.


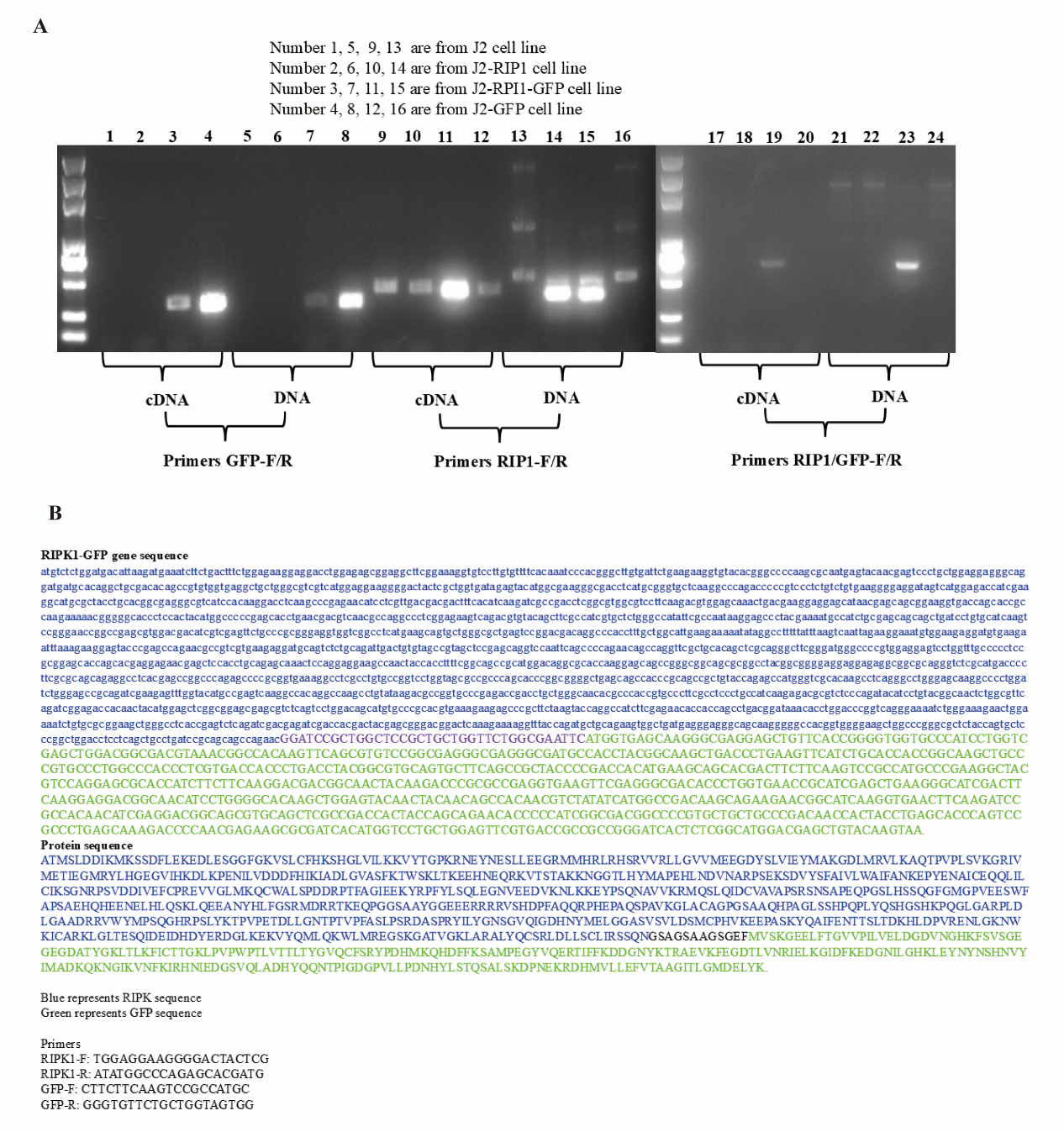


Figure S2: Validation of RIP1-GFP expression in IPEC-J2 cells using PCR and sequencing.

(A) PCR analysis of cDNA and DNA from different IPEC-J2 cell lines. Lanes 1, 5, 9, and 13 are from the J2 cell line; lanes 2, 6, 10, and 14 are from the J2-RIP1 cell line; lanes 3, 7, 11, and 15 are from the J2-RIP1-GFP cell line; and lanes 4, 8, 12, and 16 are from the J2-GFP cell line. The primers used for amplification are GFP-F/R, RIP1-F/R, and RIP1/GFP-F/R, as indicated.

(B) Sequence alignment of the RIPK1-GFP fusion gene. The GFP sequence is highlighted in green, and the RIPK1 sequence is highlighted in blue. The primers used for amplification are also listed.
